# A mechanistic model of ADHD as resulting from dopamine phasic/tonic imbalance during reinforcement learning

**DOI:** 10.3389/fncom.2022.849323

**Published:** 2022-07-18

**Authors:** Florence Véronneau-Veilleux, Philippe Robaey, Mauro Ursino, Fahima Nekka

**Affiliations:** ^1^Faculté de Pharmacie, Université de Montréal, Montreal, QC, Canada; ^2^Children's Hospital of Eastern Ontario, University of Ottawa, Ottawa, ON, Canada; ^3^Department of Electrical, Electronic and Information Engineering “Guglielmo Marconi,” University of Bologna, Bologna, Italy; ^4^Centre de Recherches Mathématiques, Université de Montréal, Montreal, QC, Canada; ^5^Centre for Applied Mathematics in Bioscience and Medicine, McGill University, Montreal, QC, Canada

**Keywords:** attention deficit hyperactivity disorder, tonic and phasic dopamine, neurocomputational model, basal ganglia, reinforcement learning

## Abstract

Attention deficit hyperactivity disorder (ADHD) is the most common neurodevelopmental disorder in children. Although the involvement of dopamine in this disorder seems to be established, the nature of dopaminergic dysfunction remains controversial. The purpose of this study was to test whether the key response characteristics of ADHD could be simulated by a mechanistic model that combines a decrease in tonic dopaminergic activity with an increase in phasic responses in cortical-striatal loops during learning reinforcement. To this end, we combined a dynamic model of dopamine with a neurocomputational model of the basal ganglia with multiple action channels. We also included a dynamic model of tonic and phasic dopamine release and control, and a learning procedure driven by tonic and phasic dopamine levels. In the model, the dopamine imbalance is the result of impaired presynaptic regulation of dopamine at the terminal level. Using this model, virtual individuals from a dopamine imbalance group and a control group were trained to associate four stimuli with four actions with fully informative reinforcement feedback. In a second phase, they were tested without feedback. Subjects in the dopamine imbalance group showed poorer performance with more variable reaction times due to the presence of fast and very slow responses, difficulty in choosing between stimuli even when they were of high intensity, and greater sensitivity to noise. Learning history was also significantly more variable in the dopamine imbalance group, explaining 75% of the variability in reaction time using quadratic regression. The response profile of the virtual subjects varied as a function of the learning history variability index to produce increasingly severe impairment, beginning with an increase in response variability alone, then accumulating a decrease in performance and finally a learning deficit. Although ADHD is certainly a heterogeneous disorder, these results suggest that typical features of ADHD can be explained by a phasic/tonic imbalance in dopaminergic activity alone.

## 1. Introduction

Attention Deficit Hyperactivity Disorder (ADHD) is a complex neurodevelopmental disorder characterized by pervasive inattention, impulsivity, and restlessness that is inconsistent with the patient's age (American Psychiatric Association, 2013). The origin of ADHD is largely genetic, and for a smaller part environmental, mostly specific to each individual (Burt, 2009; Wood et al., 2010). The first genome-genome wide meta-analysis identified twelve loci in regions containing enhancers and promoters of expression in central nervous system tissues (Demontis et al., 2019). None of these loci were linked to the dopamine system, despite the fact that dopamine genes have been associated with ADHD in candidate gene approaches (Li et al., 2006; Faraone and Larsson, 2019). Other converging evidence supports a role for dopaminergic dysfunction in ADHD. To briefly list them, most animal models used in ADHD research show some type of dopamine dysfunction (van der Kooij and Glennon, 2007). Stimulants such as methylphenidate, which are the first line of treatment, block more than 50% of dopamine transporters (DAT) in the striatum when given in therapeutic doses (Volkow et al., 1998). ADHD patients are vulnerable to drug dependence, which may be explained by an overlap of ADHD with the dopamine deficiency syndrome (Blum et al., 2008). In functional brain imaging, the most consistent findings are deficits in activity in fronto-striatal circuits where dopamine supports reinforcement learning (Dickstein et al., 2006; Norman et al., 2016). The clearest and most reproducible structural abnormalities in ADHD are located in the basal ganglia and can be normalized by the use of stimulant medications (Nakao et al., 2011). There appears to be a 5- to 10-year lag in the pruning of fronto-striatal circuits in ADHD patients compared to their typically developing peers (Dickstein, 2018). Functional magnetic resonance and diffusion tensor imaging modalities consistently indicate disrupted connectivity in regions and tracts involving fronto-striatal-thalamic loops in ADHD (Saad et al., 2020).

Different models have been proposed to account for a dopaminergic dysfunction. In the basal ganglia, dopamine release may be sustained (tonic) and regulated by prefrontal cortical afferents, or transient (phasic), caused by bursts of firing of dopaminergic neurons (Grace, 1991). The dynamic developmental theory (DDT) of ADHD proposed a hypo-dopaminergic cause. Blunted phasic dopamine bursts impair reinforcement learning (Sagvolden et al., 2005; Volkow et al., 2005), while a hypoactive tonic firing rate results in impaired extinction of previously reinforced behaviors (Sagvolden et al., 2005). A neural network developed by Frank et al. (Frank, 2005; Frank and Claus, 2006) instantiated key properties of cortico-striatal-thalamocortical loops, including direct and indirect basal ganglia pathways. These authors used this basal ganglia model to test the plausibility of the DDT of ADHD with reduced tonic and phasic dopamine levels in the striatum (Frank et al., 2007). While they showed that dopamine modulates the Go and NoGo pathways in the striatum, as well as average reaction time, they were unable to reproduce the increased variability in reaction time, a key feature of ADHD (Kofler et al., 2013), with this hypodopaminergic model alone.

As an alternative we here tested the plausibility of a model that combines a decrease in tonic dopamine activity with an increase in phasic responses (Grace, 2001). In Grace's model, this imbalance is the result of impaired presynaptic regulation of dopamine at the terminal level, and not a central decrease in DA tonic activity that is associated with other conditions, such as chronic stress (Belujon and Grace, 2015; Douma and de Kloet, 2020). This imbalance produces abnormally large reward reinforcements, which explains impulsivity, as well as the preference for smaller immediate rewards over larger delayed rewards (Jackson and MacKillop, 2016; Patros et al., 2016). This model received some support in a PET study showing reduced tonic release and increased phasic release of dopamine in the right caudate in adults with ADHD (Badgaiyan et al., 2015).

In the present study, we used a mechanistic model of the basal ganglia dopaminergic system that we previously developed to help rationally improve pharmacological interventions in Parkinson's disease (Véronneau-Veilleux et al., 2020). The model is a combination of a neurocomputational model of the basal ganglia (Baston and Ursino, 2015; Baston et al., 2016) and a model of dopamine dynamics (Dreyer, 2014) that includes dopamine release and reuptake by DAT. In addition, we included the tonic and phasic release of dopamine as well as the negative regulation of dopaminergic neuron activity by autoreceptors. We used phasic dopamine release as a reward prediction error signal (RPE) for a correct response and a phasic decrease in tonic dopamine activity as a punishment prediction error signal for a false response (Schultz, 2002). Considering that ADHD results from transactions between at-risk individuals and their specific environment (Burt, 2010; Burt et al., 2012), we used this computational model to test the hypothesis that the phasic/tonic imbalance of DA release would lead, during reinforcement learning, to the development in some individuals of ADHD characteristics, in particular response variability.

As dopamine in basal ganglia is primarily involved in learning reinforcement, we considered dopamine phasic vs. tonic release imbalance as a risk factor, and created two groups of virtual participants: one with a phasic/tonic imbalance and the other with the normal balance. We trained all of them to learn responses to 4 stimuli presented in a random sequence, using a forced-choice probabilistic task with a fixed reinforcement learning schedule and fully informative reinforcement feedback. Next, we assessed the outcome of learning reinforcement process in a test phase to determine whether or not ADHD characteristics would be present more frequently in the dopamine imbalance group than in the control group. Finally, we identified the characteristics of the learning phase that were associated with the development of these ADHD features in the dopamine imbalance group.

## 2. Methods

The mechanistic model herein developed can be divided into two parts: the dopamine dynamics model and the neurocomputational model of basal ganglia. Synaptic learning in the basal ganglia is modeled with the Hebb's rule. This rule allows the value of synaptic weights to be modified according to tonic and phasic dopamine concentrations. The simulations comprise a learning and a test phase.

### 2.1. Dopamine dynamics model

The dopamine dynamics model describes the temporal dopamine concentration, both tonic and phasic, autoreceptors occupancy and dopaminergic receptors occupancy. It was adapted from previously published models (Dreyer et al., 2010; Dreyer and Hounsgaard, 2013; Dreyer, 2014; Fuller et al., 2019).

**Figure 1:**
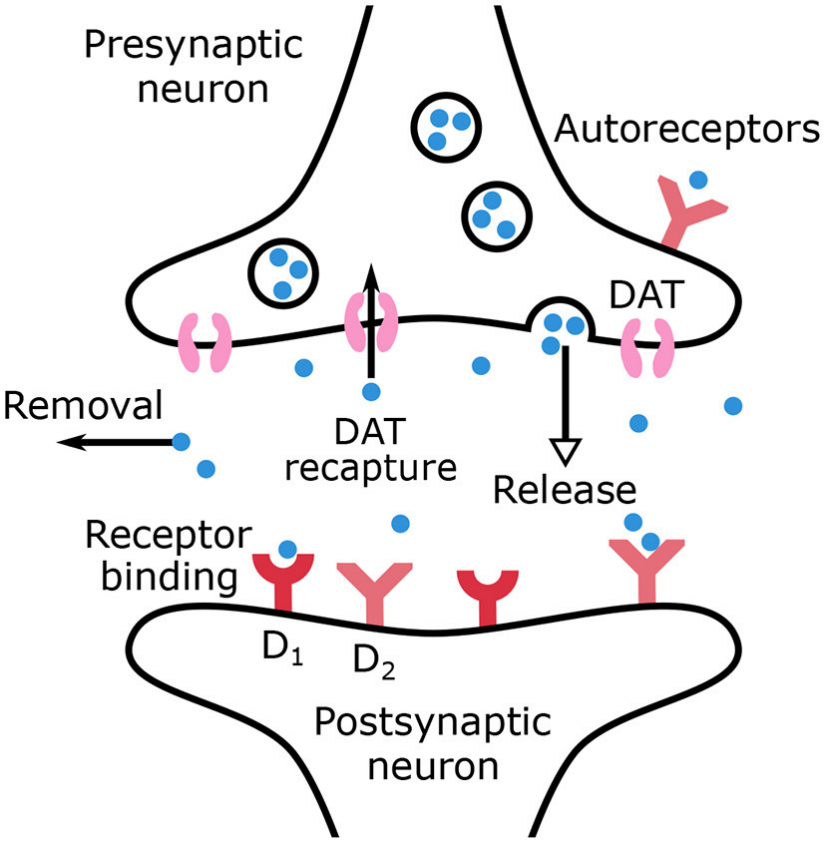
Schematic representation of dopamine release, recapture, removal and binding to receptors in the synaptic cleft.

The main mechanisms of dopamine regulation are outlined in the equations of the model and are represented in Figure 1. Dopamine is synthesized in the dopaminergic neurons and then released in the synaptic cleft. Sustained dopamine release refers to tonic dopamine, while transient dopamine release generated by bursts refers to phasic dopamine. The release of phasic dopamine is a reward prediction error signal (RPE) (Waelti et al., 2001; Marinelli and McCutcheon, 2014), whereas a drop in dopamine levels is a punishment prediction error signal. In the synaptic cleft, dopamine can be recaptured by DATs into the presynaptic neuron or be removed from the synaptic cleft by different mechanisms such as diffusion or inactivation by the Catechol-O-methyltransferase.

The remaining dopamine molecules can bind to dopaminergic autoreceptors located on the presynaptic neurons or to receptors on the postsynaptic neurons. In the present work, only dopaminergic receptors *D*_1_ and *D*_2_ are considered. All the above mentioned mechanisms are accounted for by the dopamine dynamics model, formulated in Equations (1) and (2), where *C*_*DA*_(*t*) is the dopamine concentration (μ*M*/*L*) in the synaptic cleft and *AR*(*t*) the autoreceptors occupancy.


(1) $$\begin{array}{l} \underset{\text{Dopamine concentration}}{\underset{︸}{\frac{dC_{DA}\left(t\right)}{dt}}}=\underset{\text{Dopamine Release}}{\underset{︸}{\left(I_{DA}^{tonic}+I_{DA}^{phasic}\left(t\right)\right)}}-\underset{\text{Recapture by DATs}}{\underset{︸}{\frac{V_{max}C_{DA}\left(t\right)}{\left(k_{m}+C_{DA}\left(t\right)\right)}}}\\ \;-\underset{\text{Removal}}{\underset{︸}{k_{rem}C_{DA}\left(t\right)}}, \end{array}$$

(2) $$\begin{array}{l} \underset{\text{Autoreceptor occupancy}}{\underset{︸}{\frac{dAR\left(t\right)}{dt}}}=\underset{\text{Binding to autoreceptors}}{\underset{︸}{C_{DA}\left(t\right)k_{on}\left(1-AR\left(t\right)\right)}}\\ \;-\underset{\text{Unbinding to autoreceptors}}{\underset{︸}{k_{off}AR\left(t\right)}}. \end{array}$$

As indicated in Equation (1), the release of dopamine is divided into two terms to account for both tonic and phasic release. The recapture by DATs is a saturable process described by a Michaelis-Menten equation. All other mechanisms contributing to dopamine removal are assumed to be linear (Budygin et al., 2002; Dreyer, 2014) and are schematized through the last term in the right-hand member of Equation (1). The binding to autoreceptors is proportional to dopamine concentration and free autoreceptors, while unbinding is proportional only to bound autoreceptors.

Autoreceptors have a regulatory effect on dopamine concentration. Indeed, they provide a negative feedback to adjust dopamine concentration through firing rate, synthesis, and release (Benoit-Marand et al., 2001; Beaulieu and Gainetdinov, 2011). Prolonged dopamine agonist exposure desensitizes autoreceptors in dopamine neurons (Robinson et al., 2017). Loss of inhibition influence facilitates further dopamine release and has been linked to drug abuse. Desensitization was not included in the model which is focused on the short-term effect of dopamine on autoreceptors. If tonic dopamine level decreases (in our ADHD model through increased dopamine reuptake), the temporary decrease in autoreceptor-mediated inhibition would mainly increase phasic dopamine release following the model developed by Grace (Grace, 1991, 2016). Autoinhibition of the presynaptic neurons is included in the model through the phasic release term only which is associated with the reward prediction error, while the tonic term is not here modified by autoreceptors occupancy (Grace, 1991).

The tonic dopamine release term is given by:


(3) $$\begin{array}{l} I_{DA}^{tonic}=\rho \frac{P_{r}^{tonic}n_{0}}{\alpha_{vf}N_{A}}\upsilon_{tonic}, \end{array}$$

where ρ is the terminal density, $P_{r}^{tonic}$ the tonic release probability, *n*_0_ the number of molecules released per vesicles fusion, α_*vf*_ the extracellular volume fraction, *N*_*A*_ the Avogadros constant and υ_*tonic*_ the tonic firing rate. The tonic release is independent of autoreceptors occupancy, as explained above.

The phasic release term at time *t* is given by:

- when there is no response yet, and no prediction error signal:
  (4) $$\begin{array}{l} I_{DA}^{phasic}\left(t\right)=0, \end{array}$$
- when there is a reward prediction error signal at time *t*_*reward*_:
  (5) $$\begin{array}{l} I_{DA}^{phasic}\left(t\right)=\rho \frac{\left(P_{r}^{phasic}\cdot \frac{0.334}{AR\left(t\right)}\right)n_{0}|RPE|}{\alpha_{vf}N_{A}}\left(\upsilon_{phasic}\cdot \frac{0.334}{AR\left(t\right)}\right), \end{array}$$
  (6) $$\begin{array}{l} \text{for }t_{reward}+0.1\leq t\leq t_{reward}+0.1+0.05, \end{array}$$
- when there is a punishment prediction error at time *t*_*punishment*_:
  (7) $$\begin{array}{l} C_{DA}\left(t\right)=0, \end{array}$$
  (8) $$\begin{array}{l} \text{for }t_{punishment}+0.1\leq t\leq t_{punishment}+0.1+0.05. \end{array}$$

The terminal density (ρ), the number of molecules released per vesicles fusion (*n*_0_), the extracellular volume fraction (α_*vf*_) and the Avogadros constant (*N*_*A*_) parameters are not modified by autoreceptors occupancy. Since vesicular release probability ($P_{r}^{phasic}$) and phasic firing rate (υ_*phasic*_) are decreased by autoreceptors (Grace, 1991), they are assumed to be inversely proportional to autoreceptors occupancy (Beaulieu and Gainetdinov, 2011; Dreyer and Hounsgaard, 2013). The exact relationship is not known but assumed here as inversely proportional for simplicity. The value 0.334, used to normalize the equation for the control case, corresponds to autoreceptors occupancy. Therefore, Equation (5) indicates that the activation of autoreceptors reduces phasic dopamine release. The values 0.1*s* (Bamford et al., 2018) and 0.05*s* represent the latency and duration of the reward or punishment error prediction signal, respectively. Phasic dopamine release is also proportional to the reward prediction signal (RPE). This issue will be discussed in more details in Section 2.3.

In the occurrence of a punishment, the activity of the dopamine neuron is temporarily suppressed (both tonic and phasic firing rate fall to zero). According to Equations (1) and (3), this can be simulated in the model assuming υ_*tonic*_ = 0 which corresponds to the following differential equation:


(9) $$\begin{array}{l} \frac{dC_{DA}\left(t\right)}{dt}=-\frac{V_{max}C_{DA}\left(t\right)}{k_{m}+C_{DA}\left(t\right)}-k_{rem}C_{DA}\left(t\right). \end{array}$$

With the parameters we used, this equation requires about 500 ms to reach the new equilibrium with *C*_*DA*_ = 0, which is close to the duration of dopamine neuron activity suppression after the absence of an expected reward (Schultz et al., 1997). However, the time to reach this equilibrium may vary as a function of the previous discharge rate, tonic dopamine level, or reuptake. To simplify the model, the value *C*_*DA*_ = 0 was directly applied at the same time as for the phasic dopamine discharge associated with a reward, as shown in Equations (7) and (8). Setting the dopamine concentration at zero instantaneously when a punishment occurs is a simplification of the physiologic mechanisms and the pause in the firing rate was defined as in Dreyer et al. (2010). This simplification was used since the purpose of this work was to study the behaviors in a qualitative manner. In future work, we will implement more physiologic parameters with their variability.

In the model, autoreceptors occupancy depends on the overall dopamine concentration (tonic and phasic). It could be argued that, due to diffusion, only a fraction of phasic dopamine reaches autoreceptors and thus alters the release. Simulations were performed to integrate this concentration gradient on phasic dopamine reaching autoreceptors, but the results were not significantly different (not shown here), therefore the version presented here was chosen for simplicity.

Finally, dopamine molecules can bind to dopaminergic receptors, corresponding to *D*_1_ and *D*_2_ receptors in the current work. The occupancy of receptors of type *i* ∈ {1, 2} in time is given by the following equation:


(10) $$\begin{array}{l} D_{i}\left(t\right)=\frac{B_{max}^{D_{i}}C_{DA}\left(t\right)}{k_{D}^{D_{i}}+C_{DA}\left(t\right)} \end{array}$$

where $B_{max}^{D_{i}}$ and $k_{D}^{D_{i}}$ are the maximal concentration and dissociation constant of type *i* receptors, respectively. Receptors occupancy will be used in the neurocomputational model of basal ganglia as the postsynaptic effect of dopamine on the neurons in the different neurotransmission pathways (Hille, 1992).

The parameter values for the dopamine dynamics model are given in Table 1. As mentioned in this Table, the dopaminergic terminal density was adapted. As this density is inhomogeneous (Dreyer, 2014; Fuller et al., 2019), its value was set to obtain a tonic dopamine concentration in the control group of 0.02 μ*M*/*L* as reported in the literature (Wanat et al., 2009; Hunger et al., 2020).

**Table 1:** Parameters value in the dopamine dynamic model.

**Parameters**	**Description**	**Value**	**Literature value**	**Reference**
*V*_*max*_	Maximal reuptake rate by DATs	Control : 1.2 *μM*/*Ls* Dopamine imbalance : 1.8 *μM*/*Ls*	[0.2 4.3]	May et al., 1988; Nicholson, 1995; Schönfuss et al., 2001; Fuller et al., 2019
*k*_*m*_	apparent Michaelis-Menten constant	0.15 *μM*/*L*	[0.1, 0.2]	May et al., 1988; Horn, 1990; John et al., 2006; Fuller et al., 2019
*k*_*rem*_	Removal rate	0.04 *s*^−1^	0.04	Dreyer and Hounsgaard, 2013
*k*_*on*_	On-rate for DA binding to presynaptic autoreceptors	10 *μM*^−1^*s*^−1^	10	Dreyer and Hounsgaard, 2013
*k*_*off*_	Off-rate for DA binding to presynaptic autoreceptors	0.4 *s*^−1^	0.4	Dreyer and Hounsgaard, 2013
ρ	Density of dopamine terminals in striatum	0.025 · 10^15^ terminals/L	adapted	
α_*vf*_	Volume fraction of extracellular space	0.21	[0.19, 0.22]	Syková and Nicholson, 2008
*n*_0_	Number of dopamine molecules released during vesicle fusion	3,000 molecules/terminal	3,000	Pothos et al., 1998; Dreyer, 2014
*N*_*A*_	Avogadros constant	6.02214076 · 10^23^ *M*^−1^	6.02214076 · 10^23^	
$P_{r}^{phasic}$	Vesicle release probability	0.06	[0.025, 0.15]	Dreyer and Hounsgaard, 2013
$P_{r}^{tonic}$	Vesicle release probability	0.06	[0.025, 0.15]	Dreyer and Hounsgaard, 2013
υ_*tonic*_	Average tonic firing rate	4 *s*^−1^	[4,5]	Fennell et al., 2020
υ_*phasic*_	Average phasic firing rate	40 *s*^−1^	[20,100]	Fennell et al., 2020
$B_{max}^{D1}$	*D*_1_ receptor maximal density	1.6 *μM*/*L*	1.6	Hunger et al., 2020
$k_{D}^{D1}$	*D*_1_ receptor dissociation constant	1 *μM*/*L*	1	Rice and Cragg, 2008
$B_{max}^{D2}$	*D*_2_ receptor maximal density	0.08 *μM*/*L*	0.08	Hunger et al., 2020
$k_{D}^{D2}$	*D*_2_ receptor dissociation constant	0.01 *μM*/*L*	0.01	Rice and Cragg, 2008

Using the developed model, two groups of virtual individuals were created: control and dopamine imbalance individuals. The difference between the two groups lies in the modification of the *V*_*max*_ parameter of Equation (1). From a mathematical standpoint, the parameter *k*_*m*_ could also have been decreased to obtain similar results.

### 2.2. Neurocomputational model of basal ganglia

Tonic and phasic dopamine are coding prediction error signals in the basal ganglia (Schultz, 2017). ADHD is associated with dopamine dysfunctions in the cortex and the basal ganglia (Giedd et al., 2001; Seidman et al., 2005; Nakao et al., 2011; Cubillo et al., 2012; Frodl and Skokauskas, 2012; Oldehinkel et al., 2016). Hence, a neurocomputational model of basal ganglia with a learning procedure was added to the dopamine dynamics model.

The neurocomputational model presented here is an adaptation from the model developed in Baston et al. (2016). It involves the temporal neural activity in the cortex, the thalamus and the different regions of the basal ganglia (striatum, globus pallidus pars interna and pars externa, and subthalamic nucleus), with a representation of the external stimulus *S*. The neuronal activities are normalized to obtain a value between 0 and 1. The connection between each region follows three neurotransmission pathways: direct, indirect and hyperdirect. The direct pathway promotes movement, the indirect inhibits it, and the hyperdirect pathway suppresses erroneous movements. *D*_1_ and *D*_2_ receptors occupancy have an excitatory effect in the direct pathway and an inhibitory effect in the indirect pathway, respectively. Both pathways are potentiated by the effect of cholinergic interneurons, also included in the model.

**Figure 2:**
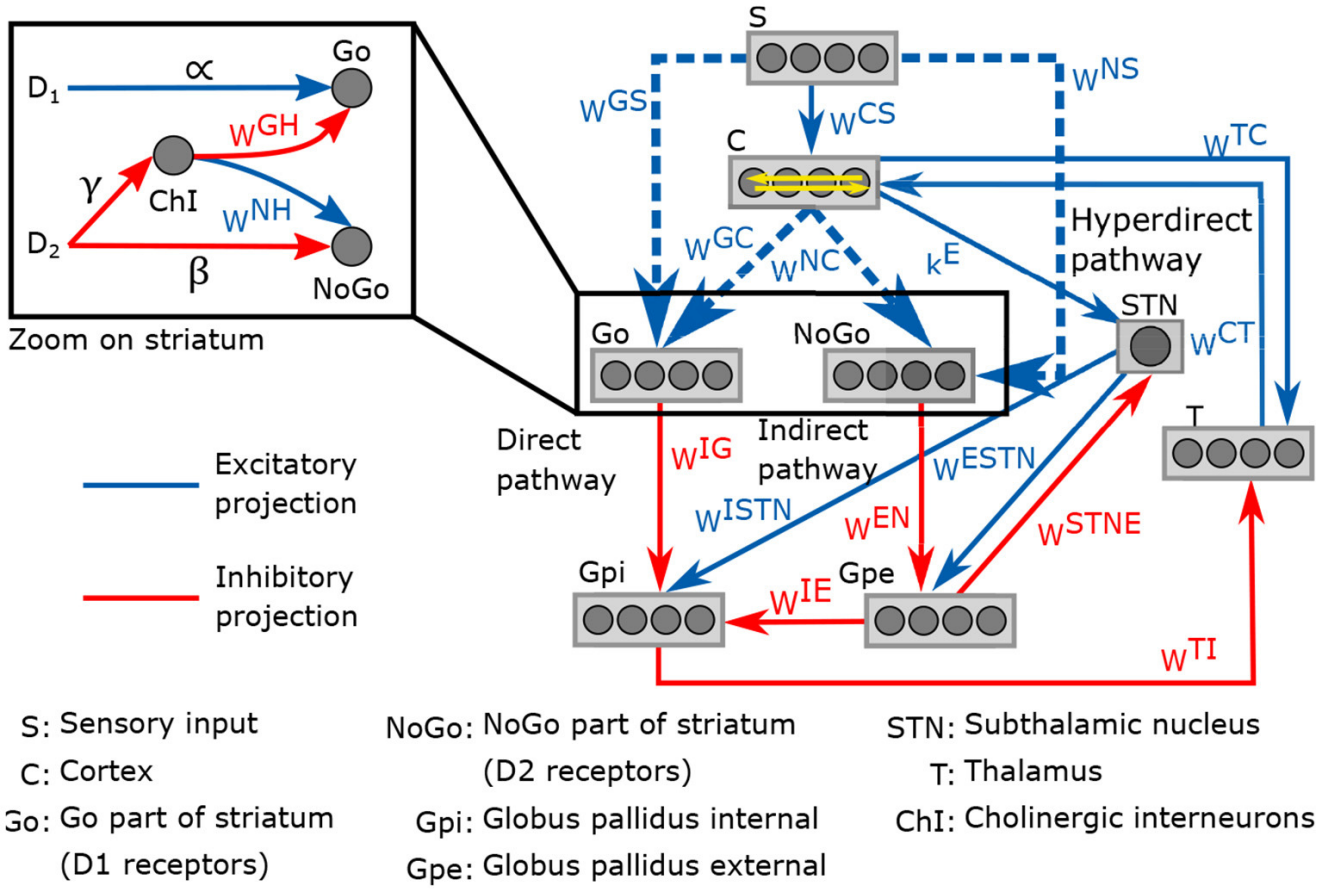
Schematic representation of the neurocomputational model of basal and its four action channels ganglia.

A representation of the neurocomputational model of basal ganglia is given in Figure 2. Each region of the model is divided into four action channels, representing different alternative choices. This division allow investigating the response of basal ganglia to various target stimuli. Neural activity in each action channel is computed through an ordinary differential equation, simulating neural dynamics, and a sigmoidal relationship, which mimics the typical non-linear phenomena of the neurons (lower threshold and upper saturation). The input to each differential equation is calculated by summing all the upstream activities converging to that neuron, weighted by the synaptic strength. The synaptic weight matrices correspond to the weight of connections between the regions for all four action channels. Equations and parameter values of the model can be found in the Supplementary Material.

### 2.3. Learning in the basal ganglia

Impairments in reinforcement learning are thought to be involved in ADHD (Sagvolden et al., 2005; Tripp and Wickens, 2008; Alexander and Farrelly, 2018). Therefore, we included a reinforcement learning process with reward and punishment prediction error signals in the model. The strength of connections between each region of basal ganglia is given by synaptic weight matrices noted *w*^*ij*^, where *i* and *j* are the postsynaptic and presynaptic regions, respectively. The values of these weights can be modified by the learning process. For simplicity, only matrices related to striatum, *w*^*GS*^, *w*^*NS*^, *w*^*GC*^, *w*^*NC*^, were considered to be plastic; these connections are represented by dashed lines in Figure 2. The matrices *w*^*GC*^ and *w*^*NC*^ are diagonal while *w*^*GS*^ and *w*^*NS*^ are full matrices. At the beginning of the learning process, these weight matrices are in a naive state, with no differentiation between the actions channels. Here are the initial value of the matrices:


(11) $$\begin{array}{l} w^{GC}=w^{NC}=\left[\begin{array}{cccc} 0.5 & 0 & 0 & 0\\ 0 & 0.5 & 0 & 0\\ 0 & 0 & 0.5 & 0\\ 0 & 0 & 0 & 0.5 \end{array}\right], \end{array}$$

(12) $$\begin{array}{l} w^{GS}=w^{NS}=\left[\begin{array}{cccc} 0.5 & 0.5 & 0.5 & 0.5\\ 0.5 & 0.5 & 0.5 & 0.5\\ 0.5 & 0.5 & 0.5 & 0.5\\ 0.5 & 0.5 & 0.5 & 0.5 \end{array}\right]. \end{array}$$

We here give the details of a typical trial of the learning process. A stimulus representation *S* is sent for 800 ms to each action channel. One channel will receive a strong stimulus of value 1, another one receives a weaker stimulus of value 0.2, while the two others receive even weaker stimuli with a value of 0.1 each. In the present work, we used an input vector with the same dimension as the number of possible actions, with a higher value (close to 1) at the same position of the rewarded action, and a smaller value at the positions of the punished actions, just to simplify the final analysis of the synapses. An input vector with different dimensions and with different values could be used as well, resulting in a more complex pattern of synapses. The idea here is to simply associate an input vector to a “winner takes all” output vector, considered as the selected response. The possible considered vectors for *S* are $S=\left[\begin{array}{cccc} 1 & 0.2 & 0.1 & 0.1 \end{array}\right]$, $S=\left[\begin{array}{cccc} 0.2 & 1 & 0.1 & 0.1 \end{array}\right]$, $S=\left[\begin{array}{cccc} 0.1 & 0.1 & 0.2 & 1 \end{array}\right]$ and $S=\left[\begin{array}{cccc} 0.1 & 0.1 & 1 & 0.2 \end{array}\right]$. Neuronal activity in all regions of basal ganglia are computed for 800 ms. An action is considered to have been performed or chosen if the activity in its related action channel in the cortex is above 0.9, while the activity in all other channels is close to zero, using the winner-takes-all dynamics implemented in the cortex.

We used a fixed scale of prediction error values throughout learning. The prediction error is the discrepancy between observed and expected outcome, and a naive subject cannot predict whether the response would be correct or not. If the chosen action is in the action channel with the highest value of *S*, a reward prediction error of 1 is attributed. If however the second highest value (0.2) is chosen, a smaller reward prediction error of 0.1 is attributed. A punishment prediction error is given when the lowest value (0.1) is chosen. Rewards prediction errors are signaled by phasic dopamine peaks governed by Equation (6). When a punishment prediction error occurs, dopamine concentration drops to zero. This is equivalent to providing the virtual subjects with rewards and punishments, but we delivered directly the reward/punishment prediction error dopamine signals. These prediction errors are the differences between received and predicted rewards (Schultz, 2016), where here the virtual patient always predicts a reward when an action is chosen. This process is repeated over 1,000 trials (epochs). Once the learning procedure is complete, the model is expected to effectively differentiate between weak and strong stimuli, so that responses occur only when strong stimuli are applied.

The resulting rewards/punishments prediction error signal will lead to a modification of the synaptic weights contained in the matrices. These weights modifications during the learning process are dictated by the Hebb Rule, which states two neurons having both high activity will strengthen their connection, whereas connection will weaken in case of neurons with opposite activity. The Hebb rule describes how much the weights are increased or decreased at each step of the training procedure. In particular, the following equation holds at each step to assign a new synaptic value, Baston and Ursino (2015):


(13) $$\begin{array}{l} w^{AB}\leftarrow w^{AB}+\Delta w^{AB}, \end{array}$$

where *w*^*AB*^ represents the matrix containing the weights from the presynaptic region *B* to the postsynaptic region *A*, with *A* being either *S* or *C* in Figure 2 and *B* being either G (Go) or N (NoGo) in the same figure, and Δ*w*^*AB*^ is the synaptic change computed at that step. Each row in these matrices represent the synapses entering the postsynaptic neuron, and each column those emerging from the presynaptic one. Hence, all matrices have 4 × 4 dimensions in the work presented here. This modification of the synaptic weights happens once every epoch between a latency period of 0.1*s* and for a duration of 0.05*s* once an action is chosen. The latency and duration are the same as the ones for the reward/punishment error prediction signal. The individual elements at position *ij* in the array Δ*w*^*AB*^ are computed through the following equation (Hebb rule):


(14) $$\begin{array}{l} \Delta w_{ij}^{AB}=\phi \cdot {\left(y_{j}^{B}-\vartheta_{presynaptic}\right)}^{+}\left(y_{i}^{A}-\vartheta_{postsynaptic}\right), \end{array}$$

where $y_{j}^{B}$ is the activity of the presynaptic neuron in the action channel *j* of the region *B*, $y_{i}^{A}$ is the activity of the postsynaptic neuron in the action channel *i* of the region *A* and ϑ_*presynaptic*_, ϑ_*postsynaptic*_ the pre- and postsynaptic thresholds. The positive part function ([]^+^) ensures that learning occurs only if the presynaptic neurons are excited and their activity is above the threshold. Dopamine is thought to have the ability to modulate synaptic plasticity, although the exact relationship does not seem to be established (Reynolds and Wickens, 2002; Frémaux and Gerstner, 2015; Madadi Asl et al., 2019). From previous work, it seemed reasonable to assume a proportional relationship with dopamine ratio and RPE. Of course, in case of diagonal matrices (*w*^*GC*^ and *w*^*NC*^), only the elements with *i* = *j* are trained, compared to non-diagonal matrices *w*^*GS*^ and *w*^*NS*^ where all elements are trained. The gain parameter ϕ is proportional to the reward prediction error since, for example, a large reward prediction error will lead to a larger variation in the synaptic value than a small reward prediction error. The gain parameter is also proportional to the ratio of phasic peak and tonic dopamine. This ratio is calculated beforehand and considered as a constant. The equation is the following:


(15) $$\begin{array}{l} \phi =0.0013\cdot |RPE|\cdot \text{DA ratio}, \end{array}$$

(16) $$\begin{array}{l} \text{DA ratio}=\left(\frac{C_{DA}^{phasic}-C_{DA}^{tonic}}{C_{DA}^{tonic}}\right). \end{array}$$

The dopamine ratio is higher in the dopamine imbalance group (with a value of ~ 8.3) compared to the control one (with a value of ~ 3), so the gain parameter ϕ is higher.

### 2.4. Simulation of virtual patients groups

The control and dopamine imbalance groups, with 10 virtual subjects each, were created with the model. The only difference between the two groups is in the value of *V*_*max*_. A higher rate of dopamine recapture is expected to lower the dopamine tonic concentration which in turn is expected to increase the phasic dopamine concentration, and thus in the tonic phasic dopamine ratio, through a lower occupancy of autoreceptor. The steps of the learning procedure of a subject are summarized below.

1. The synaptic weight matrices *w*^*GS*^, *w*^*NS*^, *w*^*GC*^, *w*^*NC*^ start in a naive state.
2. Out of the four choices ($S=\left[\begin{array}{cccc} 1 & 0.2 & 0.1 & 0.1 \end{array}\right]$, $S=\left[\begin{array}{cccc} 0.2 & 1 & 0.1 & 0.1 \end{array}\right]$, $S=\left[\begin{array}{cccc} 0.1 & 0.1 & 0.2 & 1 \end{array}\right]$ and $S=\left[\begin{array}{cccc} 0.1 & 0.1 & 1 & 0.2 \end{array}\right]$), a stimulus *S* in sent to the cortex for 800 ms. The process will be repeated for the other 3 stimuli in a random order. Noise was added in the cortex, derived from a uniform distribution and ranging from 0 to 0.2. The seed of the noise differentiates between individuals within a group but not between groups, while the *V*_*max*_ value differentiates between the two groups. For example, the control individual #1 has the same noise's seed as dopamine imbalance individual #1, but a different value of *V*_*max*_. At the end of the 800 *ms*, the subject receives either a large or a small reward prediction error signal according to his choice of the action that corresponds to the highest or the second strongest stimulus, respectively. Otherwise, the patient receives a punishment prediction error signal. Transient peaks of phasic dopamine are given accordingly and the Hebb rule is applied to modify the value of synapses. This process is repeated with the three other choices of *S*.
3. Step 2 is repeated 250 times for a total of 250 × 4 = 1, 000 epochs.
4. Once the training phase is over, the performance of the virtual subjects in each group was assessed in a testing phase. For each individual, the weight matrices were fixed to the values found at the end of the training process to assess their performance.

During the test phase, we also used a four-choice reaction time task. A series of stimuli are presented to the virtual individuals in the different action channels through a signal *S* of the neurocomputational model of BG to the cortex. The stimulus in the targeted action channel has a value of 1 with the addition of noise. Noise is also added in the other action channels directly in the cortex. Each stimulus is presented for 1, 800 *ms* with a 500 *ms* pause in between each stimulus. The criterion for a response is an activity in one of the four action channels in the cortex *C*, which constitute the output of the model, greater than 0.9. Due to the winner-takes-all dynamics, the other three channels will then have activity close to zero. For simplicity purposes, a response in the same action channel as the target stimulus is considered as a success. Otherwise it constitutes a failure. Or course successes and failures could have been defined in different ways. The idea here is simply to associate to an input vector, an output vector considered as the correct responses.

During the test phase, there is always a response after a stimulus, being a success or a failure. The number of correct answers or successes represents the performance of the virtual individuals. Each individual is presented 100 stimuli. The mean and standard deviation of the percentage of successes and of the reaction times are computed in each simulated group. Stimulus of different amplitudes were also sent in the first action channel and the responses were recorded to study the differentiation between weak and strong signals. In order to compare the ability of differentiating between weak and strong signals, we repeated the task and computed the cortex activity for different values of noise added to the input signal (*S*).

During the test phase, reaction times were also computed. The reaction time is here defined by the difference between the time at which the neuronal activity in one of the action channels reaches a value of 0.9 and the time at which the stimulus was sent in the sensory representation *S*.

## 3. Results

### 3.1. Tonic and phasic dopamine release

**Figure 3:**
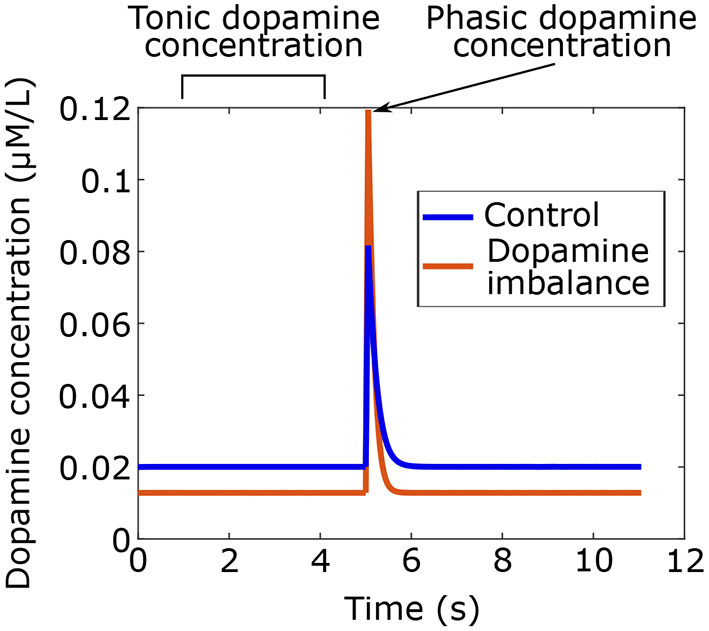
Tonic and phasic dopamine concentrations in time simulated with the model for the dopamine imbalance and the control group. In the dopamine imbalance group, tonic dopamine levels are lower due to increased recapture, which leads to decreased autoreceptor occupancy. Reduced autoreceptor occupancy causes higher peak of phasic dopamine because of autoregulation.

Using the model, dopamine concentrations were simulated for the two groups as shown in Figure 3. Phasic peaks were created by a burst lasting 0.05 s.

As seen in Figure 3, dopamine imbalance individuals have lower tonic dopamine concentration due to higher dopamine recapture. In turn, autoreceptors regulation causes higher phasic dopamine concentration. This dopamine imbalance will have different impact on the learning process in the basal ganglia.

### 3.2. Performance during the training phase

During the training phase, we computed the number of trials to obtain 5 successful responses over 10 successive trials. All participants in the normal group reached the learning criterion, but 2 participants in the dopamine imbalance group failed to do so even after 1,000 trials. The number of trials to reach criterion was on average 65.1 (*SD* = 52.6) in the control group, but 20% higher in the dopamine imbalance group, with an average of 85.5 (*SD* = 67.8), excluding those who never reached the criterion.

### 3.3. Performance during the test phase

**Figure 4:**
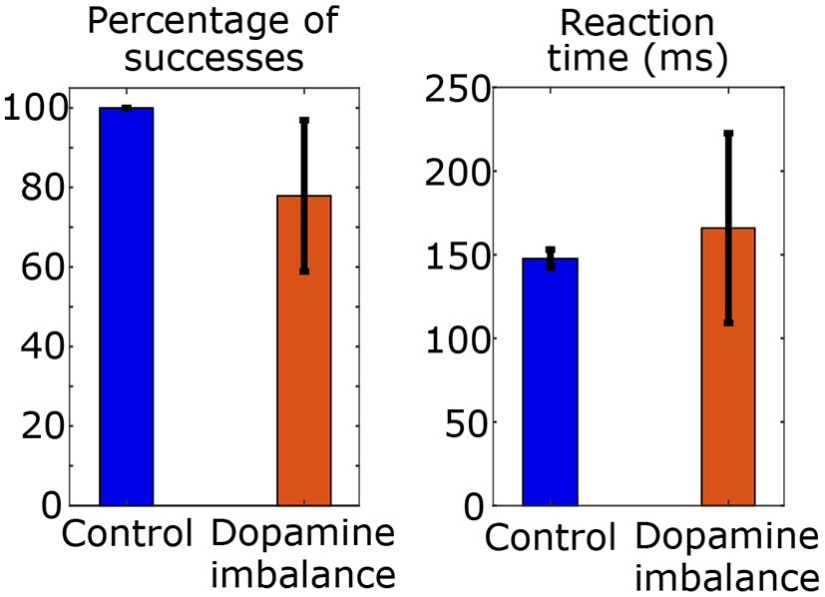
Mean and standard deviation of reaction time and percentage of success of choices in a series of 100 stimulus in each group.

In the first task, the mean and standard deviation of the percentage of successes to a series of 100 stimuli and of reaction times are computed in each simulated group and shown in Figure 4.

The mean reaction time in the control group is 148 ms and the standard deviation is 5 ms. The mean percentage of successes is 100 with a standard deviation of 0. In the dopamine imbalance group, the mean reaction time is 166 ms with a standard deviation of 57 ms. The mean percentage of successes is 78 with a standard deviation of 19. As shown in Figure 4, the rate of successes was lower and more variable in the dopamine imbalance group, as compared to the control group. Moreover, the simulated mean reaction times was slower in the dopamine imbalance group than in the control group. In our simulations, the mean and standard deviation of reaction times are respectively, 1.12 and 11.4 times larger in the dopamine imbalance group than in the control group. The significance of the reaction time difference was not evaluated because only 10 patients were simulated in this study to present the model. Also, as described further, the patients in the dopamine imbalance group are heterogeneous and can be divided into three subgroups with different mean reaction times.

**Figure 5:**
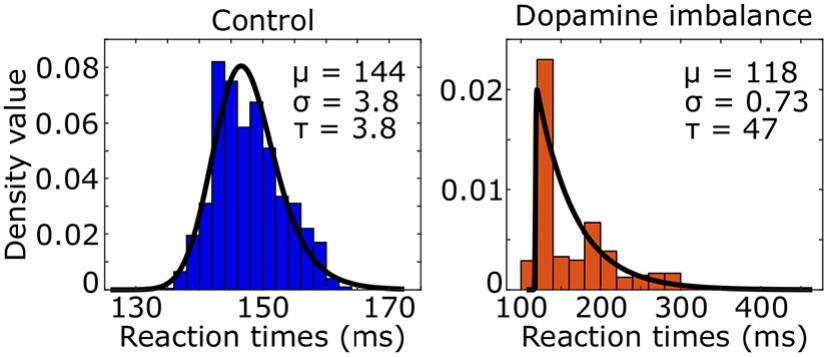
Histogram (colored boxes) and fitted density function (black line) of simulated reaction times of the virtual individuals in the control and dopamine imbalance groups.

We used the ex-Gaussian distribution to estimate the reaction time distribution by combining a normal and an exponential distribution. Three parameters characterized the ex-Gaussian distribution: the mean μ and standard deviation σ of the normal distribution, and τ representing the mean and standard deviation of the exponential part. An ex-Gaussian distribution was fitted to the simulated reaction times of the virtual individuals as seen in Figure 5.

The τ parameter was 12 times larger in the dopamine imbalance group than in the control group (47 vs. 3.8) while the μ parameter was 0.82 times smaller (118 vs. 144).

### 3.4. Performance with increasing noise

**Figure 6:**
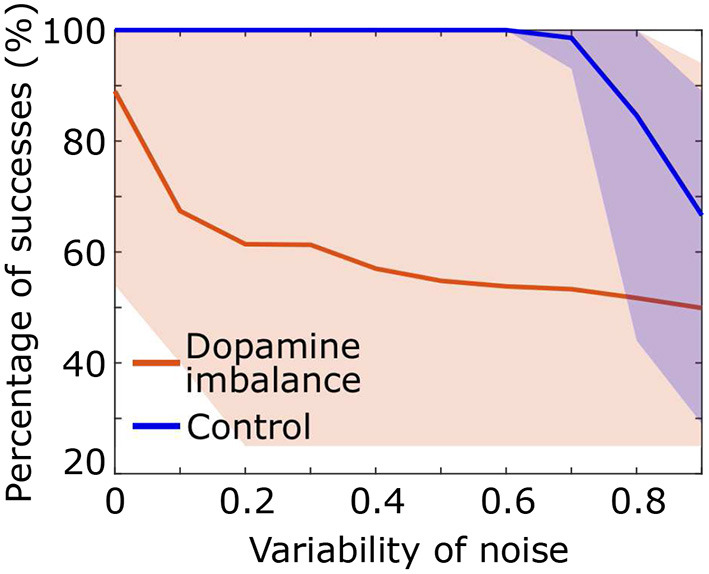
Percentage of success as a function of noise standard deviation for each individual in the dopamine imbalance group. The curve of each individual are contained in the shaded area.

We assessed the performance of the individuals in each group described in the above section by increasing the standard deviation of the noise added to the input signal *S*. A series of 100 stimuli was again presented with noise directly added to the stimulus representation in the cortex *S*, with a mean of 1 and a standard deviation ranging from 0 to 1. As the standard deviation of the noise increases, the probability of having high intensity noise increases which further complicates decision making for the virtual patients and therefore affects the percentage of successes. Figure 6 shows that in the dopamine imbalance group the mean percentage of successes (orange solid line) quickly dropped while the variability (orange shaded area) increased with increasing noise variability. By contrast, in the control group, the performance remained optimal, with no variability, until the noise variability was greater than 0.6.

### 3.5. Input and output of basal ganglia

**Figure 7:**
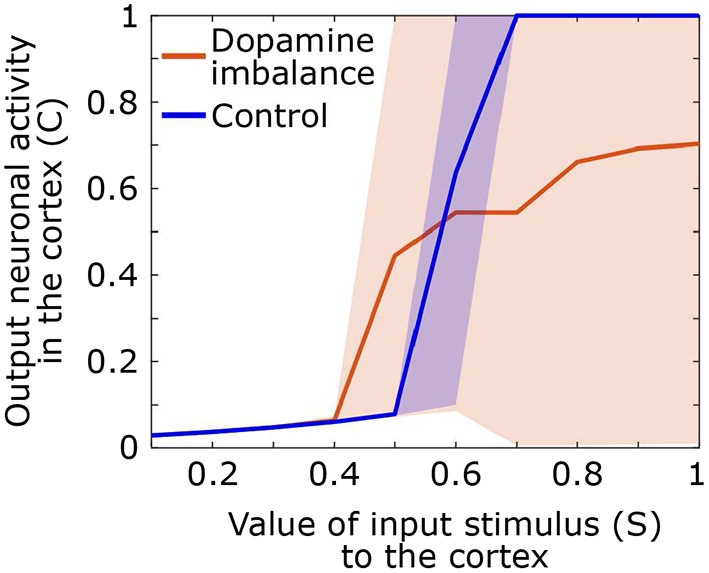
Output neuronal activity in the cortex as a function of different input stimuli. Solid line: mean neuronal activity of the individuals in each group, shaded area: 5th and 95th percentiles of neuronal activity of the individuals in the group.

During the test phase, we also computed the output activity in the cortex related to the response as a function of the input value of the stimulus. A stimulus of different amplitudes, ranging from 0.1 to 1, is sent in the first action channel while all three other channels receive noise of small amplitude. The mean, the 5th and the 95th output curves of the cortex neuronal activity in the first action channel as a function of the input signal value for each group are shown in Figure 7.

By comparing neural activity at basal ganglia input and output, it is clear that in control subjects, the basal ganglia have a high neural gain. Response-related activity is suppressed until stimulus-related cortical activity reaches 0.5 in the control group. Output activity then increases rapidly for an input between 0.5 and 0.7 at which point it remains maximal. In contrast, in the dopamine imbalance group, activity is suppressed up to an input of 0.4, after which the gain increases rapidly but only for stimulus-related activity between 0.4 and 0.5. For stimulus-related activity values between 0.5 and 1, the gain is strongly attenuated as response-related activity increases from 4.5 to 7. However, the most striking aspect of the gain is the extreme variability of the output in the dopamine imbalance group, which ranges from 0 to 1 in response to stimulus-related activity values between 0.7 and 1. In this group, some individuals respond correctly and others have wrong responses which will lead to an output activity close to zero due to the winner-takes-all dynamic, thus inducing high variability. In contrast, in the controls, the variability is almost zero, except for the amplification phase, especially around the inflection point.

### 3.6. Evolution of synaptic weights

Four synaptic weights matrices were modified during training: *w*^*GS*^, *w*^*NS*^ (stimulus-related synaptic weights) and *w*^*GC*^, *w*^*NC*^ (response-related synaptic weights). These matrices start in a naive configuration, with no differentiation between the four action channels. They are modified during the training by using the Hebb Rule, with a gain parameter that is proportional to the phasic vs. tonic dopamine ratio.

Over the course of the 1,000 trials in the training phase, the matrix weights changed differently between the two groups, and between individual subjects within each group. Indeed, the trends of synaptic weight evolution were the same for the control and dopamine imbalance groups, but inter-individual differences in synaptic weights and their evolution during learning were much larger in the dopamine imbalance group. Hence, inter-individual differences were much larger at the end of the learning phase in the dopamine imbalance than in the control group. More details on the evolution of the synaptic weight matrices are given in the Supplementary Material.

### 3.7. History of rewards and punishments prediction errors during training

In the present section, a metric is developed to differentiate the performance in the test phase of the dopamine imbalance group from the control one based on their history during the training phase. During the training process, the history of rewards and punishments is stored in a vector with value 1 for a large reward, 0.1 for a small reward, −1 for a punishment and 0 for no response. It is therefore possible to study the history of each individual and to relate it to his performance in the test phase.

**Figure 8:**
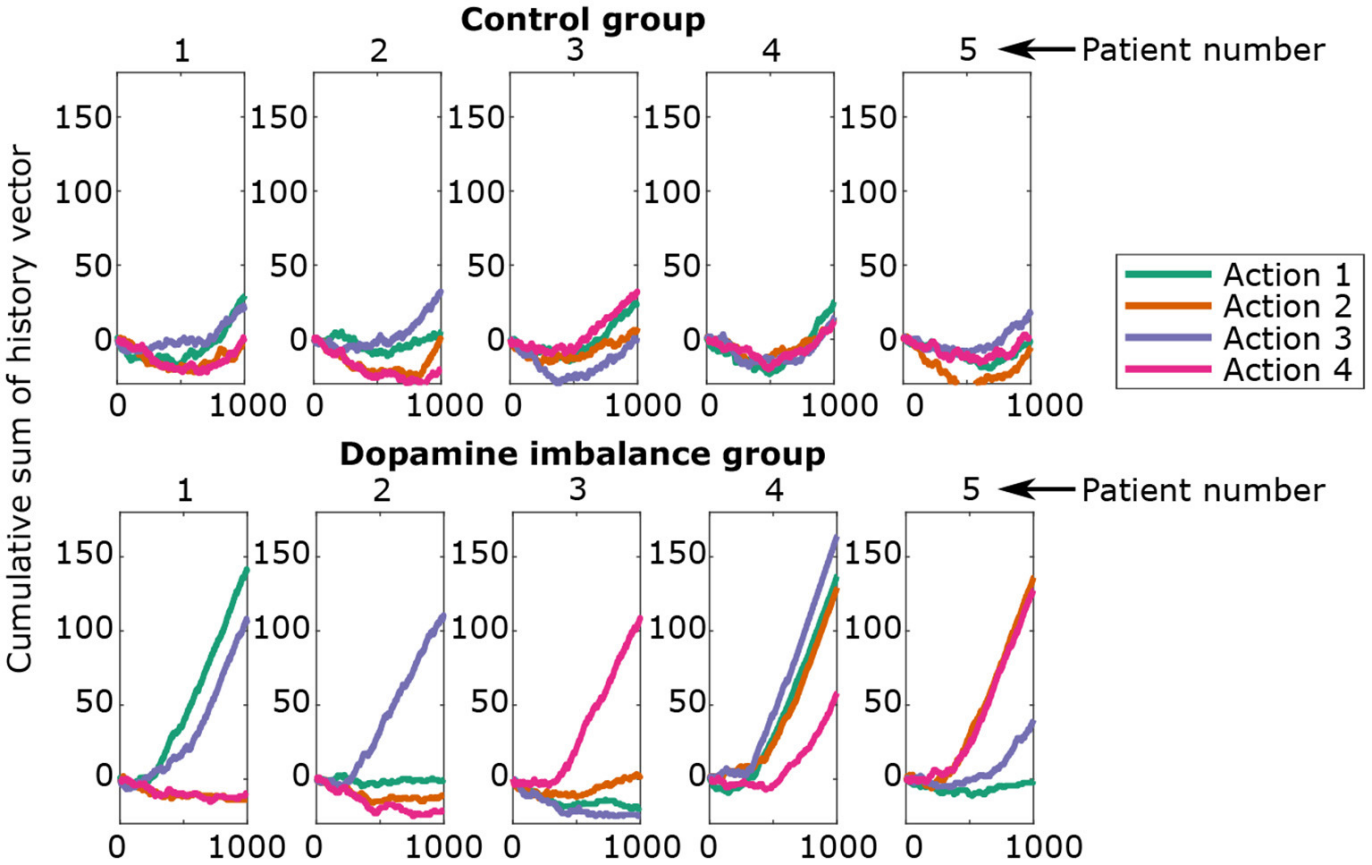
Cumulative sum of history vector at each epoch for the first five individuals in each group.

Figure 8 shows the cumulative sum of the history vector for each action channel of the first 5 individuals in each group. A negative cumulative sum results from a series of failures overcoming successes, while a positive cumulative sum would indicate the opposite.

There seems to be an initial phase in which there is an excess of errors. The virtual individuals start in a naive state, meaning no differentiation between the action channels. Hence, the initial responses have a random success rate of 25% and can lead to an excess of errors. In the second phase (> 500 epoch), rewards prediction errors dominate over punishments for all actions.

**Figure 9:**
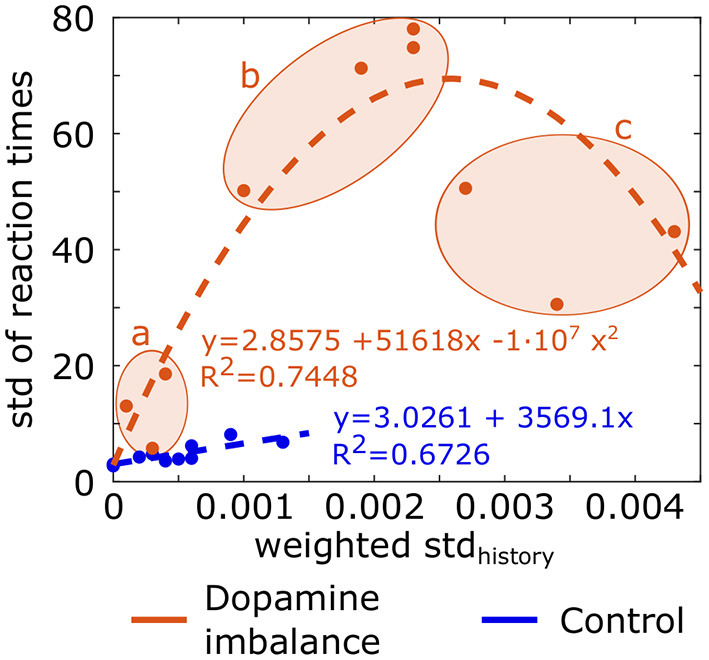
Standard deviation (std) of reaction times for each individual in each group as a function of the *weighted std*_*history*_. Equation of the linear regression and quadratic regression performed, respectively in the control group and dopamine imbalance group are shown. Individuals in the dopamine imbalance group are divided into three sub-groups, a, b, and c.

Individuals from the control group seem to learn each action in a proportional way for all action channels. The individuals in the dopamine imbalance group had a higher number of rewards for some action channels at the expense of the others. In order to quantify the inter-individual differences in learning, a weighted standard deviation (*weighted std*) for the cumulative sum of history was computed for each individual, and expressed by the following equations:


(17) $$\begin{array}{l} ratio=\frac{1}{1,000}\frac{\sum_{i=1}^{1,000}\sum_{j=1}^{4}\#\text{negative }cumsum_{action_{j}}\left(i\right)}{\sum_{i=1}^{1,000}\sum_{j=1}^{4}\#\text{positive }cumsum_{action_{j}}\left(i\right)}, \end{array}$$

(18) $$\begin{array}{l} std_{history}=\frac{1}{1,000}\sqrt{\overset{1,000}{\underset{i=1}{\sum }}\left(\overset{4}{\underset{j=1}{\sum }}{\left(cumsum_{action_{j}}\left(i\right)-mean\left(i\right)\right)}^{2}\right)}, \end{array}$$

(19) $$\begin{array}{l} weighted\;std_{history}=ratio\cdot std_{history} \end{array}$$

where *i* is the epoch number, *j* the action number, *cumsum*_*action*_*j*__ (*i*) the cumulative sum of history vector for action *j* at epoch *i* and *mean*(*i*) is the mean of cumulative history at epoch *i* for all action channels. The standard deviation of the history (*std*_*history*_) is weighted by a ratio to take into account the fact that the cumulative sum of history is either positive or negative. The ratio is the sum of negative cumulative sum of history divided by the sum of positive cumulative sum of history, leading to a larger ratio when the negative cumulative sum exceeds the positive one. Division by 1,000 is for scaling. The *weighted std*_*history*_ was larger in the dopamine imbalance group than the control one. In order to assess the relationship between the training and test phase, a plot of the standard deviation of the reaction times as a function of the *weighted std*_*history*_ value is depicted in Figure 9. A linear regression (dashed line) and a quadratic function (dashed curve) between the *weighted std*_*history*_ and the standard deviation of reaction times were applied to the control group and the imbalance group, respectively. The individuals in the dopamine imbalance group could be divided into three subgroups (a, b, and c) along the quadratic regression as seen in Figure 9. Group a contained the individuals with a perfect performance, low μ, low σ and low τ, which explains their proximity to the individuals in the control group. The individuals less than perfect performance were divided into groups b (75% of successes) and c (60% of successes). The distribution of reaction times in the group b is closer to an exponential distribution than to a normal one with low μ and σ but very high τ. These individuals have both fast and very slow reaction times, driving thus the mean to a high value. As the *weighted std*_*history*_ increases for individuals in group c, the performance further decreased with fewer correct responses, the μ parameters increased, and the σ and τ had intermediate values and were quite similar.

## 4. Discussion

In the current work, we investigated the effect of phasic vs. tonic dopamine imbalance during reinforcement learning on overt responses and on synaptic weights in the basal ganglia. We altered the phasic vs. tonic ratio by increasing the rate of maximal dopamine reuptake by DATs. As the rate of dopamine reuptake increases, the tonic level of dopamine decreases, which results in a decrease in autoreceptor binding, and in turn in an increase in the phasic response (Ford, 2014). This modification increased the phasic response by about 40%. The values of simulated dopamine concentrations that we found are consistent with those reported in the literature, with a tonic concentration between 0.005 and 0.02μ*M*/*L* (Wanat et al., 2009; Hunger et al., 2020), and a phasic concentration ranges between 0.01 and 1 μ*M*/*L* (Wickham et al., 2013). More precisely, phasic dopamine concentrations were estimated to be ~ 0.1 μ*M*/*L* in Bamford et al. (2018).

Clinically, subjects with ADHD consistently show a typical response pattern on a variety of tasks. They generally make more errors than controls and their reaction times are paradoxically both faster and slower, and more variable overall, as compared with healthy controls (Hervey et al., 2006; Huang-Pollock et al., 2012). This variability is primarily due to an excess of slow responses that can be detected by the τ component of an ex-Gaussian distribution (Kofler et al., 2013). This τ parameter best discriminates ADHD subjects (Leth-Steensen et al., 2000) from controls and appears to be a reliable endophenotype, as unaffected siblings showed intermediate values between ADHD subjects and healthy controls (Lin et al., 2015). In the present simulations, the group with dopamine imbalance also showed more variable reaction times, including an excess of very slow responses, as compared with the control group. Specifically, the μ parameter was smaller, reflecting impulsive responses, but the τ was much larger, due to a greater proportion of very slow responses, with a decrease of the σ parameter overall, which reflects the Gaussian variance. Thus, shifting the phasic/tonic dopamine ratio reproduced a response pattern typically seen in ADHD subjects, whereas a model incorporating only a decrease in both phasic and tonic dopamine release did not (Frank et al., 2007). We observed this response pattern in a simple reinforcement learning task while it has been observed in a wide variety of experimental tasks with ADHD subjects. Future studies will need to test whether this response pattern generalizes to other tasks, but it is a possibility insofar as any experimental task has a learning component. Indeed, data are typically collected after participants have reached a performance threshold during a training phase.

The change in reaction time distribution, although most typical of ADHD, is not the only difference we observed. The subjects with a dopamine imbalance also showed a lower and more variable success rate on average. Within the signal detection theory (Stanislaw and Todorov, 1999), the sensory discrimination ability is termed d'. In our simulation, the test phase used a force choice task in which d' is the percentage of successes (Stanislaw and Todorov, 1999). The control group obtained perfect results, but the success rate was decreased by 22% in the dopamine imbalance group. Subjects with ADHD also showed decreased d' in a meta-analysis of continuous performance test (CPT) performance (Huang-Pollock et al., 2012). Furthermore, we tested the effect of noise, matching each individual in the dopamine imbalance group with one individual in the control group for the seed of noise. In both groups, the success rate degraded and became more variable with increasing noise, but the dopamine imbalance group was more sensitive and showed a drop in success and a large variability for low noise levels that did not affect the performance of control subjects. Similarly, children with ADHD have been shown to have lower auditory discrimination ability than controls in the presence of background noise (Tien et al., 2019).

In order to further characterize the response pattern to stimuli of varying intensity we computed the neural gain between the input and the output of the system. A strong gain is associated with a stable attractor (Hauser et al., 2016) in which the system quickly converges to a stable activity pattern. In contrast, a weak gain is characterized by variable attractors that can lead to different unstable and shallow activity patterns. In the present simulation, for stimulus-related input values that always produced a stable response in controls (≥ 0.7), response-related output activity was much more variable in the group with dopamine imbalance. In this group, the more random responses reflected a more exploratory approach where different responses could be produced even for high stimulus-related inputs in the cortex. In experimental situations, subjects with ADHD demonstrated the same type of exploratory approach. In a probabilistic reversal learning task (Hauser et al., 2014), ADHD subjects did not choose their response strictly on the basis of their belief in the value of the stimulus, but more often took an exploratory approach. When the neural gain was estimated by a sigmoidal function, this exploratory approach also resulted in a less steep decision function. The phasic response may reinforce the response to low-intensity sensory events, which could lead to a more prolonged phase of discovery of new actions in a learning situation (Redgrave et al., 2008).

But the most significant result of the simulation, consistent with our original hypothesis, is that while all at-risk subjects had the same dopamine release imbalance, the ADHD response pattern developed to different degrees depending on the individual learning experience. On average, during this probabilistic learning task with 100% valid feedback, subjects in the dopaminergic imbalance group required more learning trials than controls to reach a success criterion. Again, this replicates a result obtained with ADHD children (Luman et al., 2020). However, the sequence of stimuli was random with a unique seed of noise for each individual within a group, which ultimately resulted in a unique learning environment for each individual within each group. This unique environment was shared with the matched individual in the other group. When we examined separately for each individual the cumulative changes in synaptic weights between cortex and basal ganglia over the course of learning, we found that individuals in the control group showed a similar history regardless of response. In contrast, in the dopamine imbalance group, individuals showed a larger increase in synaptic weight for one or more actions, with onset at different times in the first half of the training phase. As a consequence, the intraindividual differences were much larger in the dopamine imbalance group than in the control group. We computed the weighted standard deviation of the cumulative sum of history to estimate the intraindividual differences during learning. In the control group, using a linear model, we could explain 67% of the variability of individual reaction times during the test phase with the weighted cumulative sum of history. In the control group, however, we had to use a quadratic model to explain the variability between these two measures. Three subgroups of individuals could be distinguished in the dopamine imbalance group (Figure 9). Within a similar range of weighted history variability as the controls, individuals in this subgroup a showed the same perfect performance as the controls. However, the initial slope of the parabola was much steeper than in controls, reflecting the excessive reinforcement for some responses, and the variability of their reaction time was much higher than in controls, but still lower than in the rest of the dopamine imbalance group. This combination of perfect accuracy but high variability in response could define a subthreshold ADHD subgroup, where features of ADHD are already present but do not affect overt accuracy. Closer to the vertex of the parabola, we distinguish a second subgroup b of individuals with weighted history variability larger than the controls (with some negative cumulative weights), and whose accuracy was impaired though not dramatically. The distribution of reaction times contained both fast and very slow responses. Their performance most closely resembled that observed in most of the subjects diagnosed with ADHD as their functioning is clearly impaired. Individuals with extreme weighted history variability (with mostly negative cumulative weights) were hardly learned the stimulus-response association and their performance was even poorer. Their reaction time distribution looked more gaussian with a large variability and very slow mean reaction time. Individuals in this subgroup c could be compared to subjects with a severe ADHD leading to a learning disability.

In conclusion, variability in response history is much greater in subjects with dopamine imbalance, although they were exposed on average to the same learning environment as controls. Intraindividual variability in response times is related to intraindividual variability in experience with the learning environment. It increases when certain responses are reinforced at the expense of other responses during learning, making response selection more difficult in a test phase. But this variability in experience, and therefore also in response times, is much more pronounced in subjects with an imbalance in dopamine release. For subjects in subgroups a and b, the increase in response time variability as a function of weighted learning history variability is approximately linear, but the slope is much steeper than for controls. In these subjects, the increase in phasic dopamine release at the expense of tonic release can excessively strengthen or weaken cortico-striatal synapses associated with different responses and strengthen some responses at the expense of others. These imbalances lead first to an increase in response time variability with a mixture of fast and slow responses, and as these imbalances increase during learning to a decrease in performance in the test phase. In contrast, healthy controls show little variation in the history vector during learning. Consequently, they exhibited a small normal variation in reaction time that was also predicted by the weighted variability of the history with a linear function, but with a smaller slope that reflects a more balanced reinforcement of responses. To the extent that functional connectivity between the striatum and cortex reflects changes in their synaptic connections, our model is consistent with the observed correlation between inattention and hyperactivity/impulsivity scores in networks involving the striatum (Oldehinkel et al., 2016). As these changes are marked by the strengthening of some connections at the expense of others, this also explains the contradictory results in studies comparing an ADHD group with a control group that report either hypoconnectivity (Cao et al., 2009; Posner et al., 2013) or hyperconnectivity (Tian et al., 2006; Costa Dias et al., 2013) within the cortico-striato-thalamo-cortical loops in ADHD.

This qualitative agreement we observed between simulations and experimental findings is remarkable because it is achieved by altering a single parameter of dopaminergic terminal functioning, which results in phasic-tonic imbalance in dopamine release. Frank's model (Frank et al., 2007), which implemented a reduction in both phasic and tonic dopamine levels, needed to incorporate a noradrenergic component with an increased tonic vs. phasic ratio in order to mimic the increase in reaction time variability observed in ADHD subjects. These authors did not further analyze the distribution of reaction time as a function of noradrenaline release imbalance, so we do not know whether this model reproduces the typical ex-gaussian distribution that we found. Obviously, our results do not rule out noradrenergic dysfunction in ADHD. There is strong evidence of it. Drugs modulating norepinephrine transmission by blocking the NET such as atomoxetine (Schwartz and Correll, 2014) or the alpha2-adrenergic agonists such as clonidine or guanfacine (Arnsten et al., 2007) are effective treatments for ADHD. Methylphenidate significantly occupies NET at clinically relevant doses in humans (Hannestad et al., 2010) and atomoxetine showed a dose-dependent occupancy of NET in monkeys (Ding et al., 2014). NET availability was decreased in a group of adult ADHD subjects in attention-relevant regions (frontal, parietal, thalamic, cerebellar), especially in the right hemisphere (Ulke et al., 2019). The shift from exploitation to exploration behavior has been proposed to be mediated by the firing mode of norepinephrine neurons in the locus coeruleus (Aston-Jones and Cohen, 2005). However, the results of our model suggest that norepinephrine is not necessary to reproduce the typical ADHD response pattern observed in experimental reaction time tasks, which may be accounted for by a phasic/tonic imbalance in dopaminergic activity alone. This reinforces the concept of ADHD as a heterogeneous disorder, in which the same response patterns may be produced by different dysfunctions, whether or not interacting.

Grace's model locates the mechanism of phasic/tonic imbalance of dopamine release at the level of presynaptic regulation, and not at the level of neuron activity itself (Grace, 2001). In our modeling, this presynaptic imbalance may be caused by changes in DAT reuptake (Equation 1), DA removal (Equation 1), autoreceptor occupancy (Equation 2), or a combination of these factors. We chose to increase *V*_*max*_. Yet, it is known that the binding potential of DAT, like that of D2/3 receptors, decreased in adults with ADHD (Volkow et al., 2009), and increased with long-term stimulant treatment (Fusar-Poli et al., 2012; Wang et al., 2013). DAT binding potential may reflect the density of dopamine terminals, but it is also regulated over the long term by dopamine tone, decreasing when extracellular dopamine is decreased and increasing when extracellular dopamine is increased (Zahniser and Doolen, 2001). The decrease in DAT density in ADHD adults could thus be the consequence of a long-term adaptation to a chronic low tonic dopamine level, and its increase during chronic treatment related to the restoration of a higher level. Our model does not consider these long-term changes, but only evaluates the short-term effects of the dopamine release imbalance on learning. Changes in DAT binding potential in these studies (Volkow et al., 2009; Fusar-Poli et al., 2012; Wang et al., 2013) are thus not incompatible with our choice of increasing *V*_*max*_. Moreover, in Equation (1), *V*_*max*_ or *K*_*m*_ could have been modified to obtain similar results. Beyond its density, the functional dynamics of DAT (characterized by its *K*_*m*_) may be altered by other changes (such as ion dependence, or conformational balance) that may themselves be related to genetic mutations. For example, a variable number tandem repeat (VNTR) in the 3' regulatory region of the DAT gene results in two main forms (long 10R and short 9R). The10R form has been found to be associated with ADHD, at least in children and youth (Grünblatt et al., 2019), and can combine with another VNTR to produce haplotypes (Gizer et al., 2009; Franke et al., 2010), susceptible to be modulated by epigenetic factors (Xu et al., 2015; Lambacher et al., 2020; Tonelli et al., 2020). Genetic and epigenetic changes may ultimately affect DAT dynamics. Instead of increasing *V*_*max*_, we could have also increased *K*_*rem*_ in the removal part of Equation (1). Catechol-O-methyltransferase (COMT) regulates dopamine level by degrading it, mainly in the prefrontal cortex (PFC). COMT haplotypes showed different level of activity (Diatchenko et al., 2005; Nackley et al., 2006) and it has been proposed that a decrease in COMT activity in the PFC could increase firing of pyramidal neurons and glutamate transmission in basal ganglia, leading to an increase in tonic dopamine, which in turn results in a decrease in phasic dopamine (Bilder et al., 2004). However, this model has yet to be convincingly proven (Nolan et al., 2004; Rosa et al., 2010), as the association of genetic variants of COMT with ADHD (Kang et al., 2020). In our model, dopamine phasic release decreases with autoreceptor occupancy (Benoit-Marand et al., 2001). However, the interactome governing dopamine release is much more complex and includes transporters, G-protein-coupled receptors, ion channels, intracellular signaling modulators, and protein kinases. The phasic/tonic ratio of dopamine release is thus a complex trait that varies along a continuum whose regulation is still poorly understood, but where DAT plays a key role. Increasing *V*_*max*_ was not proposed as a unique cause for a complex trait such as ADHD, but rather as a means to shift the dopamine release to a more unbalanced phasic/tonic ratio that can lead to an ADHD-like phenotype through interactions with specific learning experiences. In this perspective, we believe that our model has sound biological and clinical plausibility.

The present model has limitations. Some parameters in the model might not be identifiable and the exact value of some others is not known. The values assigned to parameters is the same for all the subjects within each group and does not reflect the interindividual variability found in control and clinical groups, but support the proof-of-concept approach. The task we used does not require inhibitory processes, which will have to be tested in further studies. Also, in further studies the dysfunctions in the noradrenergic system should also be included to better simulate the pathophysiology of ADHD. Nevertheless, our model is a first step to investigate the implication of the dopaminergic system in ADHD with a mechanistic approach.

To conclude, our model opens perspectives to be used as a platform to generate and test hypothesis regarding the dopaminergic system in ADHD. The effect of medication on performance, the impact of different patterns of noise, the difference in commission and omission errors and the continuum in the severity of ADHD symptoms could be explored with this model. The effect of gradual changes in the tonic and phasic dopamine ratio will be simulated in further studies to see if the effects on the associated behavior are continuous or discontinuous with a threshold. The model could also be used to simulate a no-response task where the patient is asked to withhold the response when a certain stimulus is sent like in the go/no-go task performed in clinical practice. This modeling approach is a promising step toward the development of an integrative model of the dopaminergic system in basal ganglia for the elucidation of its associated pathologies.

## Data availability statement

The original contributions presented in the study are included in the article/Supplementary Material, further inquiries can be directed to the corresponding author.

## Author contributions

This work makes up a portion of the doctoral thesis of FV-V. FV-V, PR, MU, and FN: construction of the model and writing of the paper. FV-V: numerical simulations. All authors contributed to the article and approved the submitted version.

## Funding

FV-V received a scholarship from the Natural Sciences and Engineering Research Council (NSERC), Canada through the PGS-D program. Support was also provided by NSERC-Industrial Chair in Pharmacometrics funded by Novartis, Pfizer and Syneos, as well as FRQNT Projet d'équipe (FN).

## Conflict of interest

The authors declare that the research was conducted in the absence of any commercial or financial relationships that could be construed as a potential conflict of interest.

## Publisher's note

All claims expressed in this article are solely those of the authors and do not necessarily represent those of their affiliated organizations, or those of the publisher, the editors and the reviewers. Any product that may be evaluated in this article, or claim that may be made by its manufacturer, is not guaranteed or endorsed by the publisher.

## References

[B1] Alexander L. Farrelly N. (2018). Attending to adult adhd: a review of the neurobiology behind adult adhd. Ir. J. Psychol. Med. 35, 237–244. 10.1017/ipm.2017.78 30124185

[B2] American Psychiatric Association. (2013). Diagnostic and Statistical Manual of Mental Disorders. Paris: Elsevier Masson.

[B3] Arnsten A. F. Scahill L. Findling R. L. (2007). alpha2-adrenergic receptor agonists for the treatment of attention-deficit/hyperactivity disorder: emerging concepts from new data. J. Child Adolesc. Psychopharmacol. 17, 393–406. 10.1089/cap.2006.0098 17822336

[B4] Aston-Jones G. Cohen J. D. (2005). Adaptive gain and the role of the locus coeruleus-norepinephrine system in optimal performance. J. Comp. Neurol. 493, 99–110. 10.1002/cne.20723 16254995

[B5] Badgaiyan R. D. Sinha S. Sajjad M. Wack D. S. (2015). Attenuated tonic and enhanced phasic release of dopamine in attention deficit hyperactivity disorder. PLoS ONE 10, e0137326. 10.1371/journal.pone.0137326 26422146 PMC4589406

[B6] Bamford N. S. Wightman R. M. Sulzer D. (2018). Dopamine's effects on corticostriatal synapses during reward-based behaviors. Neuron 97, 494–510. 10.1016/j.neuron.2018.01.006 29420932 PMC5808590

[B7] Baston C. Contin M. Calandra Buonaura G. Cortelli P. Ursino M. (2016). A mathematical model of levodopa medication effect on basal ganglia in parkinson's disease: an application to the alternate finger tapping task. Front. Hum. Neurosci. 10, 280. 10.3389/fnhum.2016.00280 27378881 PMC4911387

[B8] Baston C. Ursino M. (2015). A biologically inspired computational model of basal ganglia in action selection. Comput. Intell. Neurosci. 2015, 187417. 10.1155/2015/187417 26640481 PMC4657096

[B9] Beaulieu J.-M. Gainetdinov R. R. (2011). The physiology, signaling, and pharmacology of dopamine receptors. Pharmacol. Rev. 63, 182–217. 10.1124/pr.110.002642 21303898

[B10] Belujon P. Grace A. A. (2015). Regulation of dopamine system responsivity and its adaptive and pathological response to stress. Proc. Biol. Sci. 282, 2516. 10.1098/rspb.2014.2516 25788601 PMC4389605

[B11] Benoit-Marand M. Borrelli E. Gonon F. (2001). Inhibition of dopamine release via presynaptic d2 receptors: time course and functional characteristics *in vivo*. J. Neurosci. 21, 9134–9141. 10.1523/JNEUROSCI.21-23-09134.2001 11717346 PMC6763925

[B12] Bilder R. M. Volavka J. Lachman H. M. Grace A. A. (2004). The catechol-o-methyltransferase polymorphism: relations to the tonic-phasic dopamine hypothesis and neuropsychiatric phenotypes. Neuropsychopharmacology 29, 1943–1961. 10.1038/sj.npp.1300542 15305167

[B13] Blum K. Chen A. L.-C. Braverman E. R. Comings D. E. Chen T. J. H. Arcuri V.. (2008). Attention-deficit-hyperactivity disorder and reward deficiency syndrome. Neuropsychiatr. Dis. Treat. 4, 893–918. 10.2147/NDT.S2627 19183781 PMC2626918

[B14] Budygin E. A. John C. E. Mateo Y. Jones S. R. (2002). Lack of cocaine effect on dopamine clearance in the core and shell of the nucleus accumbens of dopamine transporter knock-out mice. J. Neuroscie. 22, RC222. 10.1523/JNEUROSCI.22-10-j0002.2002 12006604 PMC6757633

[B15] Burt S. A. (2009). Rethinking environmental contributions to child and adolescent psychopathology: a meta-analysis of shared environmental influences. Psychol. Bull. 135, 608–637. 10.1037/a0015702 19586164

[B16] Burt S. A. (2010). Are there shared environmental influences on attention-deficit/hyperactivity disorder? reply to wood, buitelaar, rijsdijk, asherson, and kuntsi [corrected] (2010). Psychol. Bull. 136, 341–343. 10.1037/a0019116 20438138

[B17] Burt S. A. Larsson H. Lichtenstein P. Klump K. L. (2012). Additional evidence against shared environmental contributions to attention-deficit/hyperactivity problems. Behav. Genet. 42, 711–721. 10.1007/s10519-012-9545-y 22566176 PMC3440545

[B18] Cao X. Cao Q. Long X. Sun L. Sui M. Zhu C.. (2009). Abnormal resting-state functional connectivity patterns of the putamen in medication-naïve children with attention deficit hyperactivity disorder. Brain Res. 1303, 195–206. 10.1016/j.brainres.2009.08.029 19699190

[B19] Costa Dias T. G. Wilson V. B. Bathula D. R. Iyer S. P. Mills K. L. Thurlow B. L.. (2013). Reward circuit connectivity relates to delay discounting in children with attention-deficit/hyperactivity disorder. Eur. Neuropsychopharmacol. 23, 33–45. 10.1016/j.euroneuro.2012.10.015 23206930 PMC3581744

[B20] Cubillo A. Halari R. Smith A. Taylor E. Rubia K. (2012). A review of fronto-striatal and fronto-cortical brain abnormalities in children and adults with attention deficit hyperactivity disorder (adhd) and new evidence for dysfunction in adults with adhd during motivation and attention. Cortex 48, 194–215. 10.1016/j.cortex.2011.04.007 21575934

[B21] Demontis D. Walters R. K. Martin J. Mattheisen M. Als T. D. Agerbo E.. (2019). Discovery of the first genome-wide significant risk loci for attention deficit/hyperactivity disorder. Nat. Genet. 51, 63–75. 10.1038/s41588-018-0269-7 30478444 PMC6481311

[B22] Diatchenko L. Slade G. D. Nackley A. G. Bhalang K. Sigurdsson A. Belfer I.. (2005). Genetic basis for individual variations in pain perception and the development of a chronic pain condition. Human Mol. Genet. 14, 135–143. 10.1093/hmg/ddi013 15537663

[B23] Dickstein D. P. (2018). Paying attention to attention-deficit/hyperactivity disorder. JAMA Netw. Open 1, e181504. 10.1001/jamanetworkopen.2018.1504 30646125

[B24] Dickstein S. G. Bannon K. Castellanos F. X. Milham M. P. (2006). The neural correlates of attention deficit hyperactivity disorder: an ale meta-analysis. J. Child Psychol. Psychiatry 47, 1051–1062. 10.1111/j.1469-7610.2006.01671.x 17073984

[B25] Ding Y.-S. Naganawa M. Gallezot J.-D. Nabulsi N. Lin S.-F. Ropchan J.. (2014). Clinical doses of atomoxetine significantly occupy both norepinephrine and serotonin transports: Implications on treatment of depression and adhd. Neuroimage 86, 164–171. 10.1016/j.neuroimage.2013.08.001 23933039

[B26] Douma E. H. de Kloet E. R. (2020). Stress-induced plasticity and functioning of ventral tegmental dopamine neurons. Neurosci. Biobehav. Rev. 108, 48–77. 10.1016/j.neubiorev.2019.10.015 31666179

[B27] Dreyer J. K. (2014). Three mechanisms by which striatal denervation causes breakdown of dopamine signaling. J. Neurosci. 34, 12444–12456. 10.1523/JNEUROSCI.1458-14.2014 25209283 PMC6615501

[B28] Dreyer J. K. Herrik K. F. Berg R. W. Hounsgaard J. D. (2010). Influence of phasic and tonic dopamine release on receptor activation. J. Neurosci. 30, 14273–14283. 10.1523/JNEUROSCI.1894-10.2010 20962248 PMC6634758

[B29] Dreyer J. K. Hounsgaard J. (2013). Mathematical model of dopamine autoreceptors and uptake inhibitors and their influence on tonic and phasic dopamine signaling. J. Neurophysiol. 109, 171–182. 10.1152/jn.00502.2012 23054599

[B30] Faraone S. V. Larsson H. (2019). Genetics of attention deficit hyperactivity disorder. Mol. Psychiatry 24, 562–575. 10.1038/s41380-018-0070-0 29892054 PMC6477889

[B31] Fennell A. M. Pitts E. G. Sexton L. L. Ferris M. J. (2020). Phasic dopamine release magnitude tracks individual differences in sensitization of locomotor response following a history of nicotine exposure. Sci. Rep. 10, 173. 10.1038/s41598-019-56884-z 31932634 PMC6957501

[B32] Ford C. P. (2014). The role of d2-autoreceptors in regulating dopamine neuron activity and transmission. Neuroscience 282, 13–22. 10.1016/j.neuroscience.2014.01.025 24463000 PMC4108583

[B33] Frank M. J. (2005). Dynamic dopamine modulation in the basal ganglia: a neurocomputational account of cognitive deficits in medicated and nonmedicated parkinsonism. J. Cogn. Neurosci. 17, 51–72. 10.1162/0898929052880093 15701239

[B34] Frank M. J. Claus E. D. (2006). Anatomy of a decision: striato-orbitofrontal interactions in reinforcement learning, decision making, and reversal. Psychol. Rev. 113, 300–326. 10.1037/0033-295X.113.2.300 16637763

[B35] Frank M. J. Santamaria A. O'Reilly R. C. Willcutt E. (2007). Testing computational models of dopamine and noradrenaline dysfunction in attention deficit/hyperactivity disorder. Neuropsychopharmacology 32, 1583–1599. 10.1038/sj.npp.1301278 17164816

[B36] Franke B. Vasquez A. A. Johansson S. Hoogman M. Romanos J. Boreatti-Hümmer A.. (2010). Multicenter analysis of the slc6a3/dat1 vntr haplotype in persistent adhd suggests differential involvement of the gene in childhood and persistent adhd. Neuropsychopharmacology 35, 656–664. 10.1038/npp.2009.170 19890261 PMC3055604

[B37] Frémaux N. Gerstner W. (2015). Neuromodulated spike-timing-dependent plasticity, and theory of three factor learning rules. Front. Neural Circ. 9, 85. 10.3389/fncir.2015.00085 26834568 PMC4717313

[B38] Frodl T. Skokauskas N. (2012). Meta-analysis of structural mri studies in children and adults with attention deficit hyperactivity disorder indicates treatment effects. Acta Psychiatr. Scand. 125, 114–126. 10.1111/j.1600-0447.2011.01786.x 22118249

[B39] Fuller J. A. Burrell M. H. Yee A. G. Liyanagama K. Lipski J. Wickens J. R.. (2019). Role of homeostatic feedback mechanisms in modulating methylphenidate actions on phasic dopamine signaling in the striatum of awake behaving rats. Progr. Neurobiol. 182, 101681. 10.1016/j.pneurobio.2019.101681 31412279

[B40] Fusar-Poli P. Rubia K. Rossi G. Sartori G. Balottin U. (2012). Striatal dopamine transporter alterations in adhd: pathophysiology or adaptation to psychostimulants? a meta-analysis. Am. J. Psychiatry 169, 264–272. 10.1176/appi.ajp.2011.11060940 22294258

[B41] Giedd J. N. Blumenthal J. Molloy E. Castellanos F. X. (2001). Brain imaging of attention deficit/hyperactivity disorder. Ann. N. Y. Acad. Sci. 931, 33–49. 10.1111/j.1749-6632.2001.tb05772.x 11462751

[B42] Gizer I. R. Ficks C. Waldman I. D. (2009). Candidate gene studies of adhd: a meta-analytic review. Hum. Genet. 126, 51–90. 10.1007/s00439-009-0694-x 19506906

[B43] Grace A. A. (1991). Phasic versus tonic dopamine release and the modulation of dopamine system responsivity: a hypothesis for the etiology of schizophrenia. Neuroscience 41, 1–24. 10.1016/0306-4522(91)90196-U 1676137

[B44] Grace A. A. (2001). Psychostimulant actions on dopamine and limbic system function: Relevance to the pathophysiology and treatment of adhd, in Stimulant Drugs and ADHD: Basic and Clinical Neuroscience (Oxford: Oxford University Press), 134–157.

[B45] Grace A. A. (2016). Dysregulation of the dopamine system in the pathophysiology of schizophrenia and depression. Nat. Rev. Neurosci. 17, 524–532. 10.1038/nrn.2016.57 27256556 PMC5166560

[B46] Grünblatt E. Werling A. M. Roth A. Romanos M. Walitza S. (2019). Association study and a systematic meta-analysis of the vntr polymorphism in the 3'-utr of dopamine transporter gene and attention-deficit hyperactivity disorder. J. Neural Trans. 126, 517–529. 10.1007/s00702-019-01998-x 30923918 PMC6456487

[B47] Hannestad J. Gallezot J.-D. Planeta-Wilson B. Lin S.-F. Williams W. A. van Dyck C. H.. (2010). Clinically relevant doses of methylphenidate significantly occupy norepinephrine transporters in humans *in vivo*. Biol. Psychiatry 68, 854–860. 10.1016/j.biopsych.2010.06.017 20691429 PMC3742016

[B48] Hauser T. U. Fiore V. G. Moutoussis M. Dolan R. J. (2016). Computational psychiatry of adhd: neural gain impairments across marrian levels of analysis. Trends Neurosci. 39, 63–73. 10.1016/j.tins.2015.12.009 26787097 PMC4746317

[B49] Hauser T. U. Iannaccone R. Ball J. Mathys C. Brandeis D. Walitza S.. (2014). Role of the medial prefrontal cortex in impaired decision making in juvenile attention-deficit/hyperactivity disorder. JAMA Psychiatry 71, 1165–1173. 10.1001/jamapsychiatry.2014.1093 25142296

[B50] Hervey A. S. Epstein J. N. Curry J. F. Tonev S. Eugene Arnold L. Keith Conners C.. (2006). Reaction time distribution analysis of neuropsychological performance in an adhd sample. Child Neuropsychol. 12, 125–140. 10.1080/09297040500499081 16754533

[B51] Hille B. (1992). G protein-coupled mechanisms and nervous signaling. Neuron 9, 187–195. 10.1016/0896-6273(92)90158-A 1353972

[B52] Horn A. S. (1990). Dopamine uptake: a review of progress in the last decade. Progr. Neurobiol. 34, 387–400. 10.1016/0301-0082(90)90033-D 2192393

[B53] Huang-Pollock C. L. Karalunas S. L. Tam H. Moore A. N. (2012). Evaluating vigilance deficits in adhd: a meta-analysis of cpt performance. J. Abnorm Psychol. 121, 360–371. 10.1037/a0027205 22428793 PMC3664643

[B54] Hunger L. Kumar A. Schmidt R. (2020). Abundance compensates kinetics: similar effect of dopamine signals on d1 and d2 receptor populations. J. Neurosci. 40, 2868–2881. 10.1523/JNEUROSCI.1951-19.2019 32071139 PMC7117896

[B55] Jackson J. N. S. MacKillop J. (2016). Attention-deficit/hyperactivity disorder and monetary delay discounting: A meta-analysis of case-control studies. Biol. Psychiatry 1, 316–325. 10.1016/j.bpsc.2016.01.007 27722208 PMC5049699

[B56] John C. E. Budygin E. A. Mateo Y. Jones S. R. (2006). Neurochemical characterization of the release and uptake of dopamine in ventral tegmental area and serotonin in substantia nigra of the mouse. J. Neurochem. 96, 267–282. 10.1111/j.1471-4159.2005.03557.x 16300629

[B57] Kang P. Luo L. Peng X. Wang Y. (2020). Association of val158met polymorphism in comt gene with attention-deficit hyperactive disorder: an updated meta-analysis. Medicine 99, e23400. 10.1097/MD.0000000000023400 33235119 PMC7710242

[B58] Kofler M. J. Rapport M. D. Sarver D. E. Raiker J. S. Orban S. A. Friedman L. M.. (2013). Reaction time variability in adhd: a meta-analytic review of 319 studies. Clin. Psychol. Rev. 33, 795–811. 10.1016/j.cpr.2013.06.001 23872284

[B59] Lambacher G. Pascale E. Pucci M. Mangiapelo S. D'Addario C. Adriani W. (2020). Search for an epigenetic biomarker in adhd diagnosis, based on the dat1 gene 5'-utr methylation: a new possible approach. Psychiatry Res. 291, 113154. 10.1016/j.psychres.2020.113154 32554184

[B60] Leth-Steensen C. Elbaz Z. K. Douglas V. I. (2000). Mean response times, variability, and skew in the responding of adhd children: a response time distributional approach. Acta Psychol. 104, 167–190. 10.1016/S0001-6918(00)00019-6 10900704

[B61] Li D. Sham P. C. Owen M. J. He L. (2006). Meta-analysis shows significant association between dopamine system genes and attention deficit hyperactivity disorder (adhd). Hum. Mol. Genet. 15, 2276–2284. 10.1093/hmg/ddl152 16774975

[B62] Lin H.-Y. Hwang-Gu S.-L. Gau S. S.-F. (2015). Intra-individual reaction time variability based on ex-gaussian distribution as a potential endophenotype for attention-deficit/hyperactivity disorder. Acta Psychiatr. Scand. 132, 39–50. 10.1111/acps.12393 25612058

[B63] Luman M. Janssen T. W. P. Bink M. van Mourik R. Maras A. Oosterlaan J. (2020). Probabilistic learning in children with attention-deficit/hyperactivity disorder. J. Attent. Disord. 25, 1407–1416. 10.1177/1087054720905094 32064998 PMC8273841

[B64] Madadi Asl M. Vahabie A. H. Valizadeh A. (2019). Dopaminergic modulation of synaptic plasticity, its role in neuropsychiatric disorders, and its computational modeling. Basic Clin. Neurosci. 10, 1–12. 10.32598/bcn.9.10.125 31031889 PMC6484184

[B65] Marinelli M. McCutcheon J. E. (2014). Heterogeneity of dopamine neuron activity across traits and states. Neuroscience 282, 176–197. 10.1016/j.neuroscience.2014.07.034 25084048 PMC4312268

[B66] May L. J. Kuhr W. G. Wightman R. M. (1988). Differentiation of dopamine overflow and uptake processes in the extracellular fluid of the rat caudate nucleus with fast-scan in vivo voltammetry. J. Neurochem. 51, 1060–1069. 10.1111/j.1471-4159.1988.tb03069.x 2971098

[B67] Nackley A. G. Shabalina S. A. Tchivileva I. E. Satterfield K. Korchynskyi O. Makarov S. S.. (2006). Human catechol-o-methyltransferase haplotypes modulate protein expression by altering mrna secondary structure. Science 314, 1930–1933. 10.1126/science.1131262 17185601

[B68] Nakao T. Radua J. Rubia K. Mataix-Cols D. (2011). Gray matter volume abnormalities in adhd: voxel-based meta-analysis exploring the effects of age and stimulant medication. Am. J. Psychiatry 168, 1154–1163. 10.1176/appi.ajp.2011.11020281 21865529

[B69] Nicholson C. (1995). Interaction between diffusion and michaelis-menten uptake of dopamine after iontophoresis in striatum. Biophys. J. 68, 1699–1715. 10.1016/S0006-3495(95)80348-6 7612814 PMC1282074

[B70] Nolan K. A. Bilder R. M. Lachman H. M. Volavka J. (2004). Catechol o-methyltransferase val158met polymorphism in schizophrenia: differential effects of val and met alleles on cognitive stability and flexibility. Am. J. Psychiatry 161, 359–361. 10.1176/appi.ajp.161.2.359 14754787

[B71] Norman L. J. Carlisi C. Lukito S. Hart H. Mataix-Cols D. Radua J.. (2016). Structural and functional brain abnormalities in attention-deficit/hyperactivity disorder and obsessive-compulsive disorder: a comparative meta-analysis. JAMA Psychiatry 73, 815–825. 10.1001/jamapsychiatry.2016.0700 27276220

[B72] Oldehinkel M. Beckmann C. F. Pruim R. H. R. van Oort E. S. B. Franke B. Hartman C. A.. (2016). Attention-deficit/hyperactivity disorder symptoms coincide with altered striatal connectivity. Biol. Psychiatry 1, 353–363. 10.1016/j.bpsc.2016.03.008 27812554 PMC5087803

[B73] Patros C. H. G. Alderson R. M. Kasper L. J. Tarle S. J. Lea S. E. Hudec K. L. (2016). Choice-impulsivity in children and adolescents with attention-deficit/hyperactivity disorder (adhd): a meta-analytic review. Clin. Psychol. Rev. 43, 162–174. 10.1016/j.cpr.2015.11.001 26602954

[B74] Posner J. Rauh V. Gruber A. Gat I. Wang Z. Peterson B. S. (2013). Dissociable attentional and affective circuits in medication-naïve children with attention-deficit/hyperactivity disorder. Psychiatry Res. 213, 24–30. 10.1016/j.pscychresns.2013.01.004 23664625 PMC3717483

[B75] Pothos E. N. Davila V. Sulzer D. (1998). Presynaptic recording of quanta from midbrain dopamine neurons and modulation of the quantal size. J. Neurosci. 18, 4106–4118. 10.1523/JNEUROSCI.18-11-04106.1998 9592091 PMC6792796

[B76] Redgrave P. Gurney K. Reynolds J. (2008). What is reinforced by phasic dopamine signals? Brain Res. Rev. 58, 322–339. 10.1016/j.brainresrev.2007.10.007 18055018

[B77] Reynolds J. N. J. Wickens J. R. (2002). Dopamine-dependent plasticity of corticostriatal synapses. Neural Netw. 15, 507–521. 10.1016/s0893-6080(02)00045-x 12371508

[B78] Rice M. E. Cragg S. J. (2008). Dopamine spillover after quantal release: rethinking dopamine transmission in the nigrostriatal pathway. Brain Res. Rev. 58, 303–313. 10.1016/j.brainresrev.2008.02.004 18433875 PMC2879278

[B79] Robinson B. G. Bunzow J. R. Grimm J. B. Lavis L. D. Dudman J. T. Brown J.. (2017). Desensitized d2 autoreceptors are resistant to trafficking. Sci. Rep. 7, 4379. 10.1038/s41598-017-04728-z 28663556 PMC5491503

[B80] Rosa E. C. Dickinson D. Apud J. Weinberger D. R. Elvevåg B. (2010). Comt val158met polymorphism, cognitive stability and cognitive flexibility: an experimental examination. Behav. Brain Funct. 6, 53. 10.1186/1744-9081-6-53 20836853 PMC2945991

[B81] Saad J. F. Griffiths K. R. Korgaonkar M. S. (2020). A systematic review of imaging studies in the combined and inattentive subtypes of attention deficit hyperactivity disorder. Front. Integr. Neurosci. 14, 31. 10.3389/fnint.2020.00031 32670028 PMC7327109

[B82] Sagvolden T. Johansen E. B. Aase H. Russell V. A. (2005). A dynamic developmental theory of attention-deficit/hyperactivity disorder (adhd) predominantly hyperactive/impulsive and combined subtypes. Behav. Brain Sci. 28, 397–419; discussion 419-68. 10.1017/S0140525X05000075 16209748

[B83] Schönfuss D. Reum T. Olshausen P. Fischer T. Morgenstern R. (2001). Modelling constant potential amperometry for investigations of dopaminergic neurotransmission kinetics *in vivo*. J. Neurosci. Methods 112, 163–172. 10.1016/S0165-0270(01)00465-4 11716951

[B84] Schultz W. (2002). Getting formal with dopamine and reward. Neuron 36, 241–263. 10.1016/S0896-6273(02)00967-4 12383780

[B85] Schultz W. (2016). Dopamine reward prediction error coding. Dial. Clin. Neurosci. 18, 23–32. 10.31887/DCNS.2016.18.1/wschultz 27069377 PMC4826767

[B86] Schultz W. (2017). Reward prediction error. Curr. Biol. 27, 369-R371. 10.1016/j.cub.2017.02.064 28535383

[B87] Schultz W. Dayan P. Montague P. R. (1997). A neural substrate of prediction and reward. Science 275, 1593–1599. 10.1126/science.275.5306.1593 9054347

[B88] Schwartz S. Correll C. U. (2014). Efficacy and safety of atomoxetine in children and adolescents with attention-deficit/hyperactivity disorder: results from a comprehensive meta-analysis and metaregression. J. Am. Acad. Child Adolesc. Psychiatry 53, 174–187. 10.1016/j.jaac.2013.11.005 24472252

[B89] Seidman L. J. Valera E. M. Makris N. (2005). Structural brain imaging of attention-deficit/hyperactivity disorder. Biol. Psychiatry 57, 1263–1272. 10.1016/j.biopsych.2004.11.019 15949998

[B90] Stanislaw H. Todorov N. (1999). Calculation of signal detection theory measures. Behav. Res. Methods Instrument. Comput. 31, 137–149. 10.3758/BF03207704 10495845

[B91] Syková E. Nicholson C. (2008). Diffusion in brain extracellular space. Physiol. Rev. 88, 1277–1340. 10.1152/physrev.00027.2007 18923183 PMC2785730

[B92] Tian L. Jiang T. Wang Y. Zang Y. He Y. Liang M.. (2006). Altered resting-state functional connectivity patterns of anterior cingulate cortex in adolescents with attention deficit hyperactivity disorder. Neurosci. Lett. 400, 39–43. 10.1016/j.neulet.2006.02.022 16510242

[B93] Tien Y.-M. Chen V. C.-H. Lo T.-S. Hsu C.-F. Gossop M. Huang K.-Y. (2019). Deficits in auditory sensory discrimination among children with attention-deficit/hyperactivity disorder. Eur. Child Adolescent Psychiatry 28, 645–653. 10.1007/s00787-018-1228-7 30229307

[B94] Tonelli E. Pascale E. Troianiello M. D'Addario C. Adriani W. (2020). Dat1 gene methylation as an epigenetic biomarker in attention deficit hyperactivity disorder: a commentary. Front. Genet. 11, 444. 10.3389/fgene.2020.00444 32477403 PMC7232962

[B95] Tripp G. Wickens J. R. (2008). Research review: dopamine transfer deficit: a neurobiological theory of altered reinforcement mechanisms in adhd. J. Child Psychol. Psychiatry 49, 691–704. 10.1111/j.1469-7610.2007.01851.x 18081766

[B96] Ulke C. Rullmann M. Huang J. Luthardt J. Becker G.-A. Patt M.. (2019). Adult attention-deficit/hyperactivity disorder is associated with reduced norepinephrine transporter availability in right attention networks: a (s,s)-o-[, javax.xml.bind.jaxbelement@32a363f0, c]methylreboxetine positron emission tomography study. Transl. Psychiatry 9, 301. 10.1038/s41398-019-0619-y 31732713 PMC6858438

[B97] van der Kooij M. A. Glennon J. C. (2007). Animal models concerning the role of dopamine in attention-deficit hyperactivity disorder. Neurosci. Biobehav. Rev. 31, 597–618. 10.1016/j.neubiorev.2006.12.002 17316796

[B98] Véronneau-Veilleux F. Robaey P. Ursino M. Nekka F. (2020). An integrative model of parkinson's disease treatment including levodopa pharmacokinetics, dopamine kinetics, basal ganglia neurotransmission and motor action throughout disease progression. J. Pharmacokinet. Pharmacodyn. 48, 133–148. 10.1007/s10928-020-09723-y 33084988

[B99] Volkow N. D. Wang G.-J. Fowler J. S. Ding Y.-S. (2005). Imaging the effects of methylphenidate on brain dopamine: new model on its therapeutic actions for attention-deficit/hyperactivity disorder. Biol. Psychiatry 57, 1410–1415. 10.1016/j.biopsych.2004.11.006 15950015

[B100] Volkow N. D. Wang G. J. Fowler J. S. Gatley S. J. Logan J. Ding Y. S.. (1998). Dopamine transporter occupancies in the human brain induced by therapeutic doses of oral methylphenidate. Am. J. Psychiatry 155, 1325–1331. 10.1176/ajp.155.10.1325 9766762

[B101] Volkow N. D. Wang G. J. Kollins S. H. Wigal T. L. Newcorn J. H. Telang F.. (2009). Evaluating dopamine reward pathway in adhd: clinical implications. JAMA 302, 1084–1091. 10.1001/jama.2009.1308 19738093 PMC2958516

[B102] Waelti P. Dickinson A. Schultz W. (2001). Dopamine responses comply with basic assumptions of formal learning theory. Nature 412, 43–48. 10.1038/35083500 11452299

[B103] Wanat M. J. Willuhn I. Clark J. J. Phillips P. E. M. (2009). Phasic dopamine release in appetitive behaviors and drug addiction. Curr. Drug Abuse Rev. 2, 195–213. 10.2174/1874473710902020195 19630749 PMC2877500

[B104] Wang G.-J. Volkow N. D. Wigal T. Kollins S. H. Newcorn J. H. Telang F.. (2013). Long-term stimulant treatment affects brain dopamine transporter level in patients with attention deficit hyperactive disorder. PLoS ONE 8, e63023. 10.1371/journal.pone.0063023 23696790 PMC3655054

[B105] Wickham R. J. Solecki W. Rathbun L. R. Neugebauer N. M. Wightman R. M. Addy N. A. (2013). Advances in studying phasic dopamine signaling in brain reward mechanisms. Front. Biosci. 5, 678. 10.2741/E678 23747914 PMC3725633

[B106] Wood A. C. Buitelaar J. Rijsdijk F. Asherson P. Kuntsi J. (2010). Rethinking shared environment as a source of variance underlying attention-deficit/hyperactivity disorder symptoms: comment on burt (2009). Psychol. Bull. 136, 331–340. 10.1037/a0019048 20438137 PMC3713551

[B107] Xu Y. Chen X.-T. Luo M. Tang Y. Zhang G. Wu D.. (2015). Multiple epigenetic factors predict the attention deficit/hyperactivity disorder among the chinese han children. J. Psychiatr. Res. 64, 40–50. 10.1016/j.jpsychires.2015.03.006 25840828

[B108] Zahniser N. R. Doolen S. (2001). Chronic and acute regulation of na+/cl−-dependent neurotransmitter transporters: drugs, substrates, presynaptic receptors, and signaling systems. Pharmacol. Therapeut. 92, 21–55. 10.1016/s0163-7258(01)00158-9 11750035

