# Supplementary material for: A mechanistic model of ADHD as resulting from dopamine phasic/tonic imbalance during reinforcement learning
Source: Front Comput Neurosci. 2022 Jul 18;16:849323. doi: 10.3389/fncom.2022.849323 (PMC9342605; doi:10.3389/fncom.2022.849323)
Supplement: Supplementary file 1 [file Data_Sheet_1.PDF]

Supplementary Material

METHODS

Neurocomputational model of basal ganglia

The equations of the neurocomputational model describing the neuronal activity in each subregions of the basal ganglia is described in this section. Abbreviations for the name of each subregions are used and described in Table S1.

Table S1. Abbreviations for the subregions of the basal ganglia

Subregion's name	Abbreviation
Sensory input	S
Cortex	C
Cortex's lateral inhibition	L
Go part of striatum (D1 receptors)	G
NoGo part of striatum (D2) receptors	N
Globus pallidus internal	I
Globus pallidus external	E
Subthalamic nucleus	STN
Thalamus	T
Cholinergic interneurons	H

The equations describing the neuronal activity in each region are of type firing rate neurons. There are four neurons in each subregions to represent the four action channels except for the subthalamic nucleus and for the cholinergic interneuron. Let i be the number of the postsynaptic neuron in the subregion k receiving current from the presynaptic neuron number j in the subregion m with a weight of w^{km}. The general form of the equations are the following:

$$\tau \frac{u_i^k}{dt} = -u_i^k + \sum_{j=1}^{4} w_{ij}^{km} y_j^m. \tag{S1}$$

The post synaptic variable u_i^k is transformed into the activity of the neuron (scaled between zero and one) by the following sigmoidal function:

$$\begin{aligned} y_i^k &= \zeta(u_i^k), \\ &= \frac{1}{1+e^{-a(u_i^k - u_0)}}. \end{aligned} \tag{S2}$$

The value of the parameters of the sigmoïdal function are presented in Table S2.

Here are the equations for the neuronal activity in each subregions:

Table S2. Values of the parameters of the sigmoïdal function

Parameter	Description	Value	Reference
a	Parameter affecting the central slope	4	[5]
u_0	Value to obtain a y_i^k of 0.5	1	

Cortical neurons:

$$\tau_L \frac{du^L}{dt} = -u^L + Ly^C, \tag{S3}$$

$$\tau \frac{du^C}{dt} = -u^C + w^{CS}S + u^L + w^{CT}y^T, \tag{S4}$$

$$y_i^C = \zeta(u_i^C)\ i \in \{1,2,3,4\}, \tag{S5}$$

Go pathway:

$$\tau \frac{u^G}{dt} = -u^G + w^{GS}S + w^{GC}y^C + \alpha \cdot D_1(y^G - \upsilon^G) + w^{GH}y^H \begin{bmatrix}1\\1\\1\\1\end{bmatrix}, \tag{S6}$$

$$y_i^G = \zeta(u_i^G)\ i \in \{1,2,3,4\}, \tag{S7}$$

NoGo pathway:

$$\tau \frac{du^N}{dt} = -u^N + w^{NS}S + w^{NC}y^C + \beta \cdot D_2 + w^{NH}y^H \begin{bmatrix}1\\1\\1\\1\end{bmatrix}, \tag{S8}$$

$$y_i^N = \zeta(u_i^N)\ i \in \{1,2,3,4\}, \tag{S9}$$

GPe neurons:

$$\tau \frac{du^E}{dt} = -u^E + w^{EN}y^N + w^{ESTN}y^{STN} + I^E, \tag{S10}$$

$$y_i^E = \zeta(u_i^E)\ i \in \{1,2,3,4\}, \tag{S11}$$

GPi neurons:

$$\tau \frac{du^I}{dt} = -u^I + w^{IG}y^G + w^{IE}y^E + w^{ISTN}y^{STN} \begin{bmatrix}1\\1\\1\\1\end{bmatrix} + I^I, \tag{S12}$$

$$y_i^I = \zeta(u_i^I)\ i \in \{1,2,3,4\}, \tag{S13}$$

STN neuron:

$$\tau \frac{du^{STN}}{dt} = -u^{STN} + k_E E + \sum_{i=1}^{4} w_i^{STNE} y_i^E, \tag{S14}$$

$$\text{with } E = y_1^C y_2^C + y_1^C y_3^C + y_1^C y_4^C + y_2^C y_3^C + y_2^C y_4^C + y_3^C y_4^C, \tag{S15}$$

$$y^{STN} = \zeta(u^{STN}), \tag{S16}$$

Thalamus neurons:

$$\tau \frac{du^T}{dt} = -u^T + w^{TI} y^I + w^{TC} y^C, \tag{S17}$$

$$y_i^T = \zeta(u_i^T) \; i \in \{1, 2, 3, 4\}, \tag{S18}$$

Cholinergic interneuron:

$$\tau \frac{du^H}{dt} = -u^H + I^H + \gamma \cdot D_2, \tag{S19}$$

$$y^H = \zeta(u^H). \tag{S20}$$

See [2, 3] for a more detailed description of the model. The weights of the connections between each subregions and membrane time constants are presented in Table S3.

The globus pallidus (intern and extern) [4] and the cholinergic interneurons [1] are tonically active, therefore there is an external input to these neurons. The value of these external inputs are presented in Table S4.

The value of the weight of dopamine effect of the Go, NoGo and cholinergic interneurons are defined in Table S5.

Evolution of synaptic weight matrices

Over the course of the 1000 trials in the training phase, the matrix weights changed differently between the two groups, and between individual subjects within each group.

Figures S1 to S8 show the matrix values across trials for the 10 subjects in each of the control and dopmaine imbalance groups. In the direct pathway, for control subjects, all stimulus-related synaptic weights first decreased, then increased slightly for successes (diagonal), but continued to decrease for errors. Response-related synaptic weights increased slightly. Inter-individual differences remained small, with a slight increase at the end of learning. In the indirect pathway, all stimulus- and response-related synaptic weights decreased, again with a slight increase in interindividual differences.

In comparison, the trends were the same for the group with dopamine imbalance, but inter-individual differences in synaptic weights and their evolution during learning were much larger. In the direct pathway, stimulus-related synaptic weights strongly increased for successes at different times during learning until they reached a maximum in some individuals, whereas they decreased slightly in others. Synaptic weights off the diagonal (errors) decreased for all individuals but diverged at different times. Synaptic weights related to the response showed the same changes as stimulus-related weights. In the indirect pathway, synaptic weights decreased for both the response and stimulus channels, but this decrease began at different

Table S3. Values of the synaptic weights and of the membrane time constants (τ) in the neurocomputational model of basal ganglia

Parameter	Description	Value	Reference
w^{CS}	Weight between S and C	$\begin{bmatrix} 1 & 1 & 1 & 1 \\ 1 & 1 & 1 & 1 \\ 1 & 1 & 1 & 1 \\ 1 & 1 & 1 & 1 \end{bmatrix}$	
L	Lateral inhibition weight	$\begin{bmatrix} 0 & -1.2 & -1.2 & -1.2 \\ -1.2 & 0 & -1.2 & -1.2 \\ -1.2 & -1.2 & 0 & -1.2 \\ -1.2 & -1.2 & -1.2 & 0 \end{bmatrix}$	
w^{CT}	Weight between T and C	$\begin{bmatrix} 4 & 0 & 0 & 0 \\ 0 & 4 & 0 & 0 \\ 0 & 0 & 4 & 0 \\ 0 & 0 & 0 & 4 \end{bmatrix}$	
w^{EN}	Weight between NoGo and GPe	$\begin{bmatrix} -2.2 & 0 & 0 & 0 \\ 0 & -2.2 & 0 & 0 \\ 0 & 0 & -2.2 & 0 \\ 0 & 0 & 0 & -2.2 \end{bmatrix}$	
w^{IE}	Weight between GPe and GPi	$\begin{bmatrix} -3 & 0 & 0 & 0 \\ 0 & -3 & 0 & 0 \\ 0 & 0 & -3 & 0 \\ 0 & 0 & 0 & -3 \end{bmatrix}$	[5]
w^{IG}	Weight between Go and GPi	$\begin{bmatrix} -36 & 0 & 0 & 0 \\ 0 & -36 & 0 & 0 \\ 0 & 0 & -36 & 0 \\ 0 & 0 & 0 & -36 \end{bmatrix}$	
w^{TC}	Weight between C and T	$\begin{bmatrix} 3 & 0 & 0 & 0 \\ 0 & 3 & 0 & 0 \\ 0 & 0 & 3 & 0 \\ 0 & 0 & 0 & 3 \end{bmatrix}$	
w^{TI}	Weight between GPi and T	$\begin{bmatrix} -3 & 0 & 0 & 0 \\ 0 & -3 & 0 & 0 \\ 0 & 0 & -3 & 0 \\ 0 & 0 & 0 & -3 \end{bmatrix}$	
k_E	Energy weight in STN	7	
w^{STNE}	Weight between GPe and STN	-1	
w^{ESTN}	Weight between STN and GPe	1	
w^{ISTN}	Weight between STN and GPi	30	
w^{GH}	Weight between ChI and Go	-1	
w^{NH}	Weight between ChI and NoGo	1	
τ	Time constant	$15\ ms$	
τ_L	Time constant for lateral inhibition	$75\ ms$	

times during learning across individuals. Hence, inter-individual differences were much larger at the end of the learning phase in the dopamine imbalance than in the control group.

Table S4. Values of the external input to the neurons

Parameter	Description	Value	Reference
I^E	External input to GPe	$\begin{bmatrix}1\\1\\1\\1\end{bmatrix}$	[5]
I^I	External input to GPi	$\begin{bmatrix}2.5\\2.5\\2.5\\2.5\end{bmatrix}$	Modified from [5]
I^H	External input to ChI	1	[5]

Table S5. Parameters value related to dopamine effect in the basal ganglia

Parameter	Description	Value	Reference
υ^G	Threshold of Go neurons	0.35	[5]
α	Dopamine weight to Go neurons	20	Modified from [5]
β	Dopamine weight to NoGo neurons	-12.5	Modified from [5]
γ	Dopamine weight to ChI	-6.25	Modified from [5]

REFERENCES

[1]Teemu Aitta-Aho, Benjamin U Phillips, Elpiniki Pappa, Y Audrey Hay, Fiona Harnischfeger, Christopher J Heath, Lisa M Saksida, Tim J Bussey, and John Apergis-Schoute. Accumbal cholinergic interneurons differentially influence motivation related to satiety signaling. *eNeuro*, 4, 2017.

[2]Chiara Baston, Manuela Contin, Giovanna Calandra Buonaura, Pietro Cortelli, and Mauro Ursino. A mathematical model of levodopa medication effect on basal ganglia in parkinson's disease: An application to the alternate finger tapping task. *Frontiers in Human Neuroscience*, 10, jun 2016.

[3]Chiara Baston and Mauro Ursino. A biologically inspired computational model of basal ganglia in action selection. *Computational Intelligence and Neuroscience*, 2015:1–24, 2015.

[4]R Mark Richardson, Curt R Freed, Shoichi A Shimamoto, and Philip A Starr. Pallidal neuronal discharge in parkinson's disease following intraputamenal fetal mesencephalic allograft. *Journal of neurology, neurosurgery, and psychiatry*, 82:266–271, March 2011.

[5]Mauro Ursino and Chiara Baston. Aberrant learning in parkinson's disease: A neurocomputational study on bradykinesia. *The European journal of neuroscience*, 47:1563–1582, June 2018.

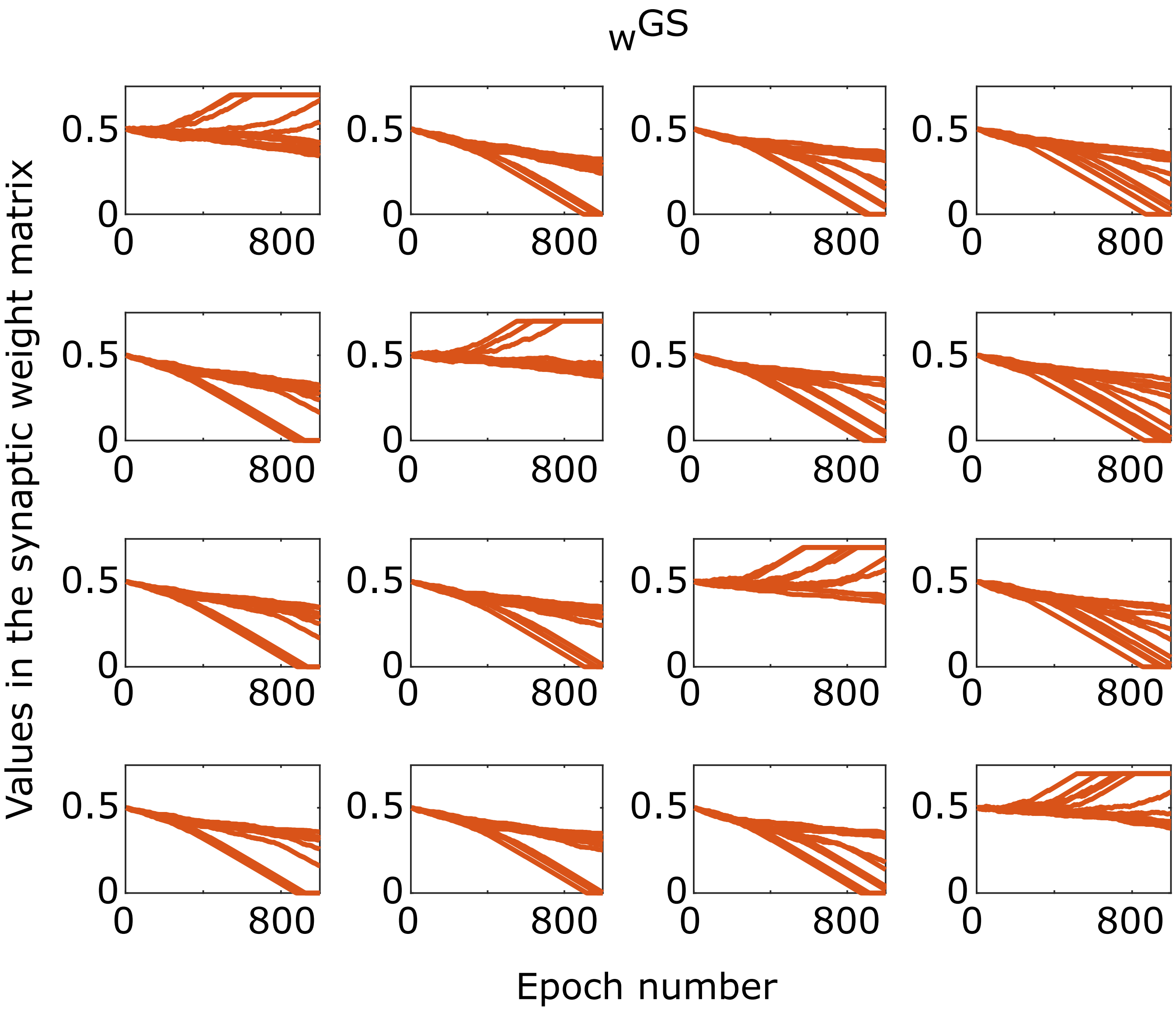

Figure S1. Evolution of the values in the w^{GS} matrix of each individual in the dopamine imbalance group

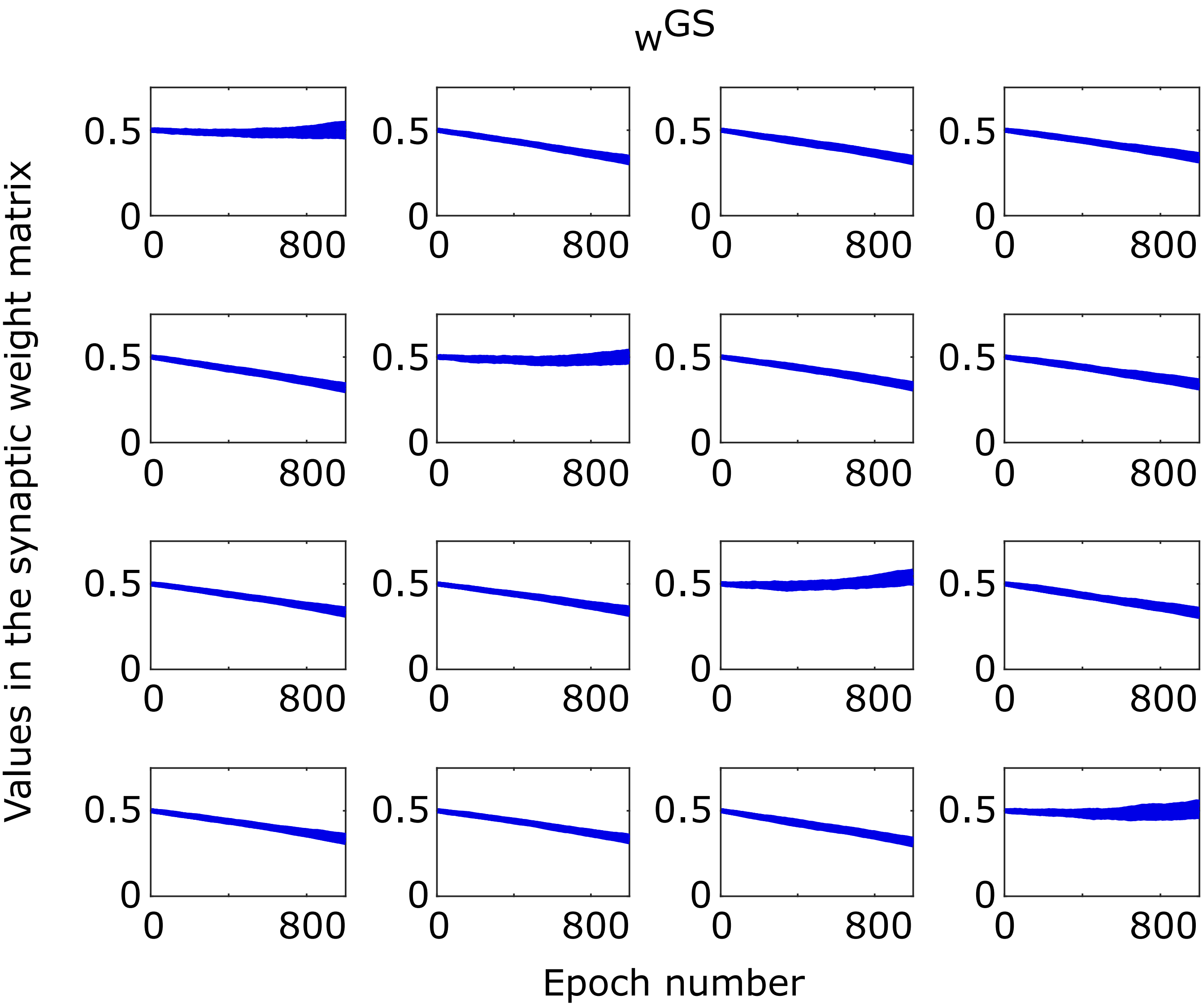

Figure S2. Evolution of the values in the w^{GS} matrix of each individual in the control group

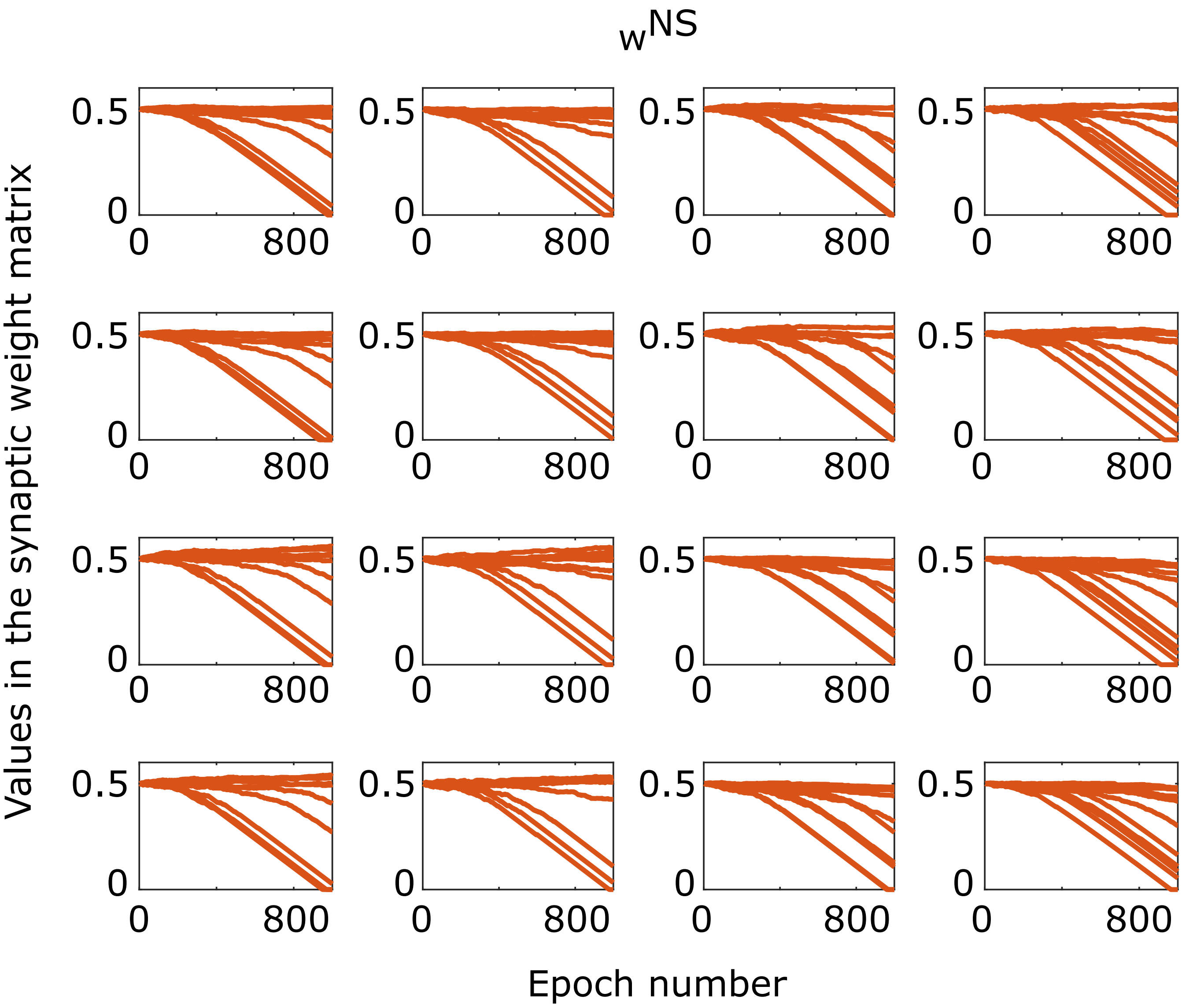

Figure S3. Evolution of the values in the w^{NS} matrix of each individual in the dopamine imbalance group group

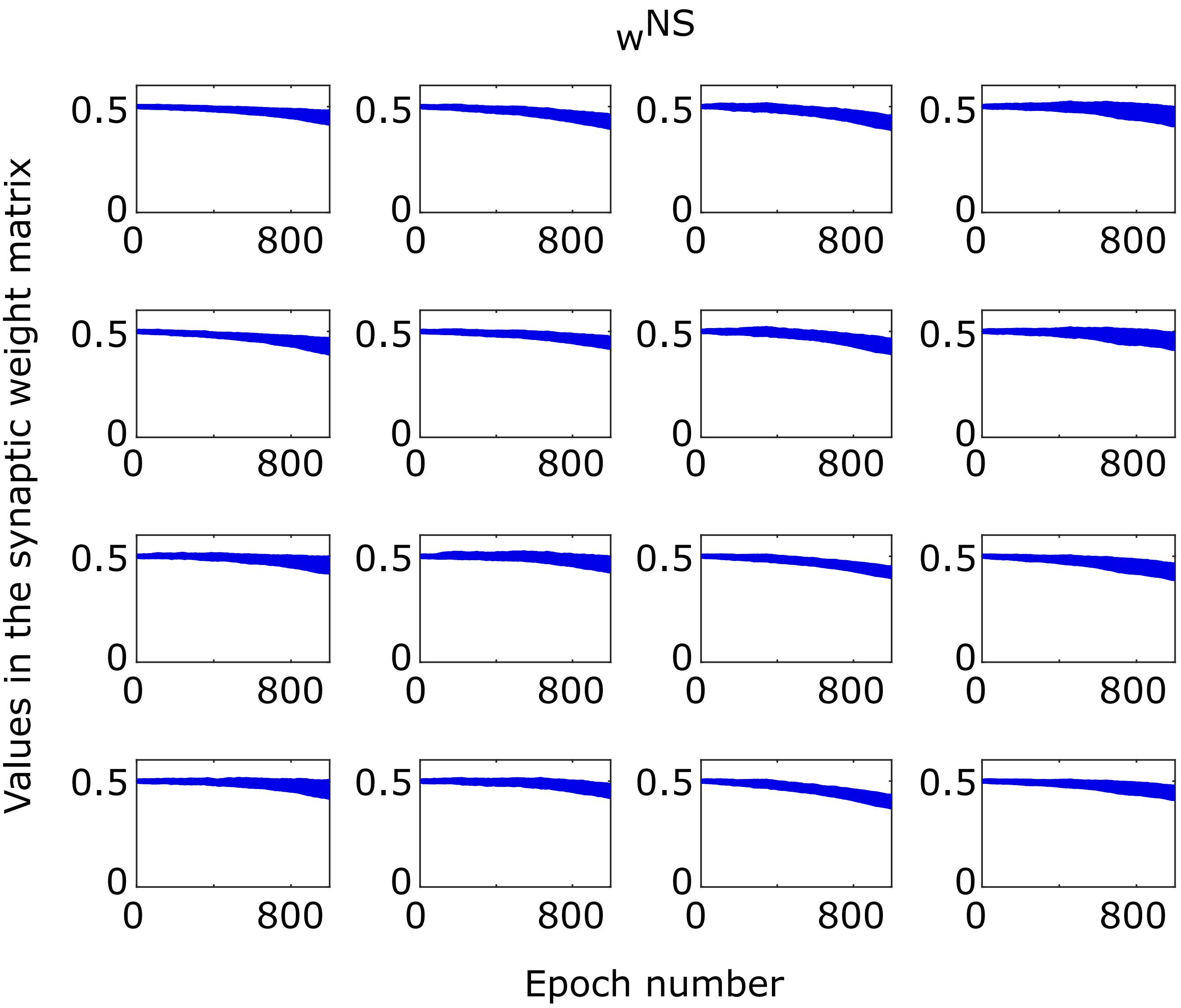

Figure S4. Evolution of the values in the w^{NS} matrix of each individual in the control group

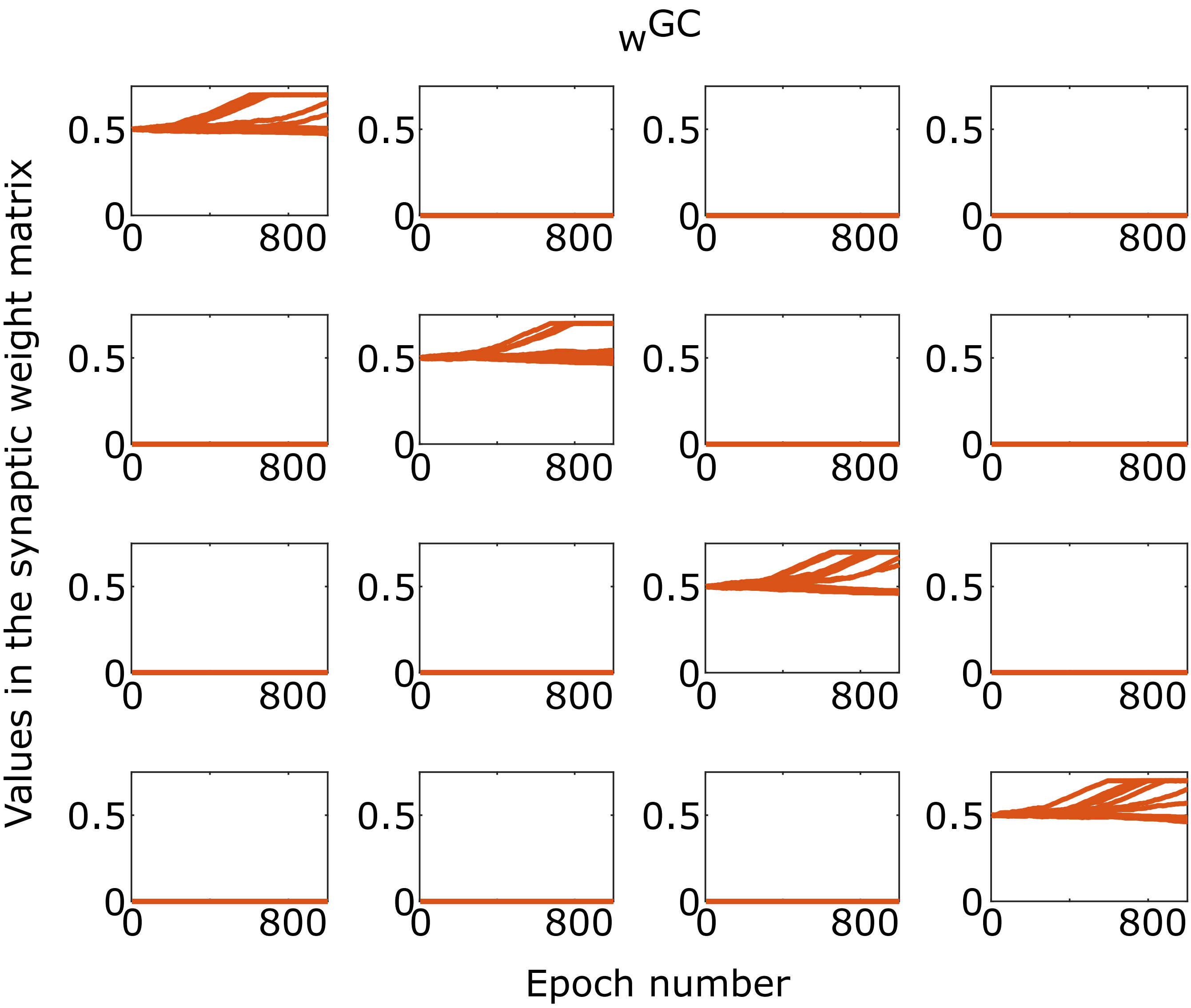

Figure S5. Evolution of the values in the w^{GC} matrix of each individual in the dopamine imbalance group

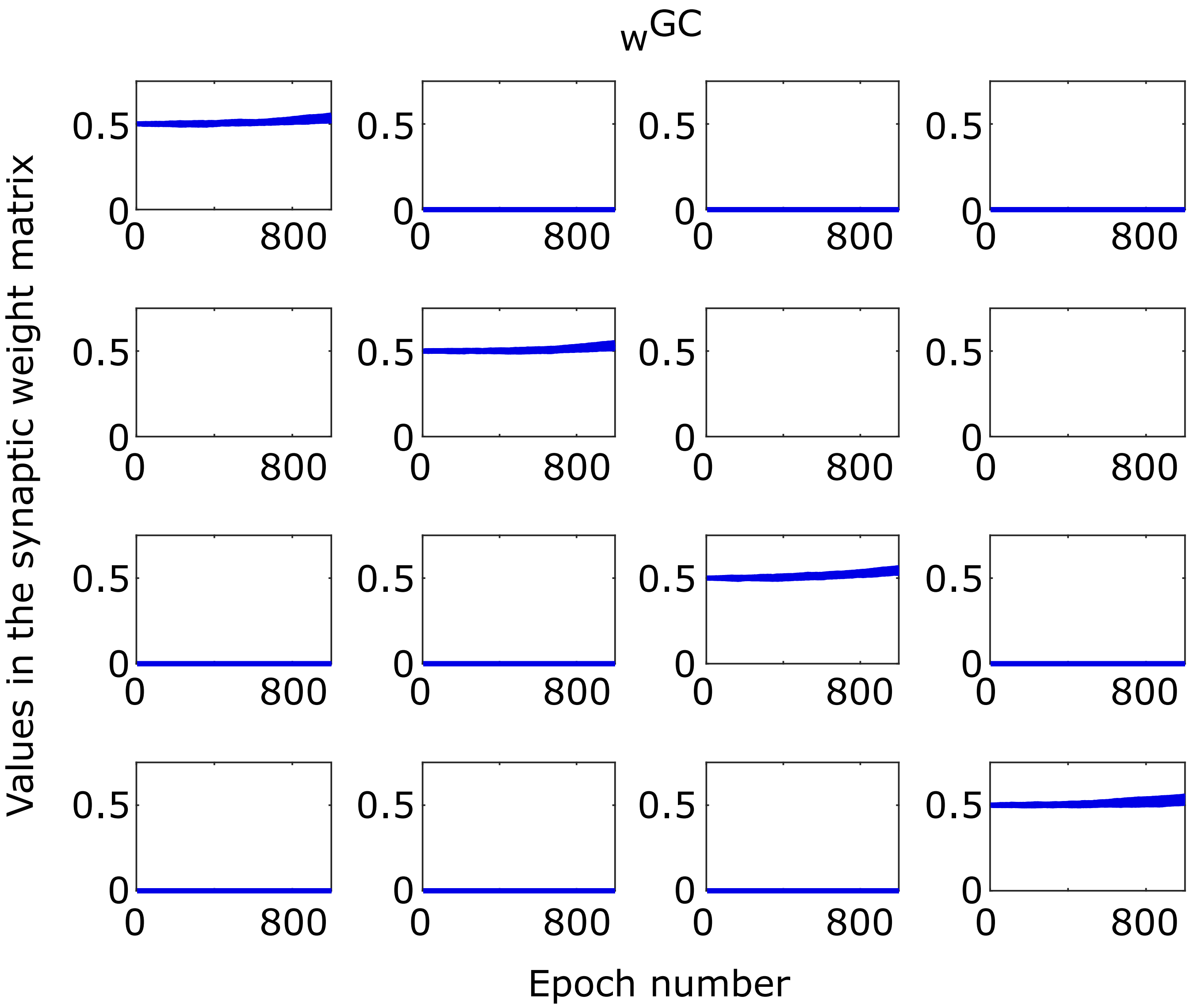

Figure S6. Evolution of the values in the w^{GC} matrix of each individual in the control group

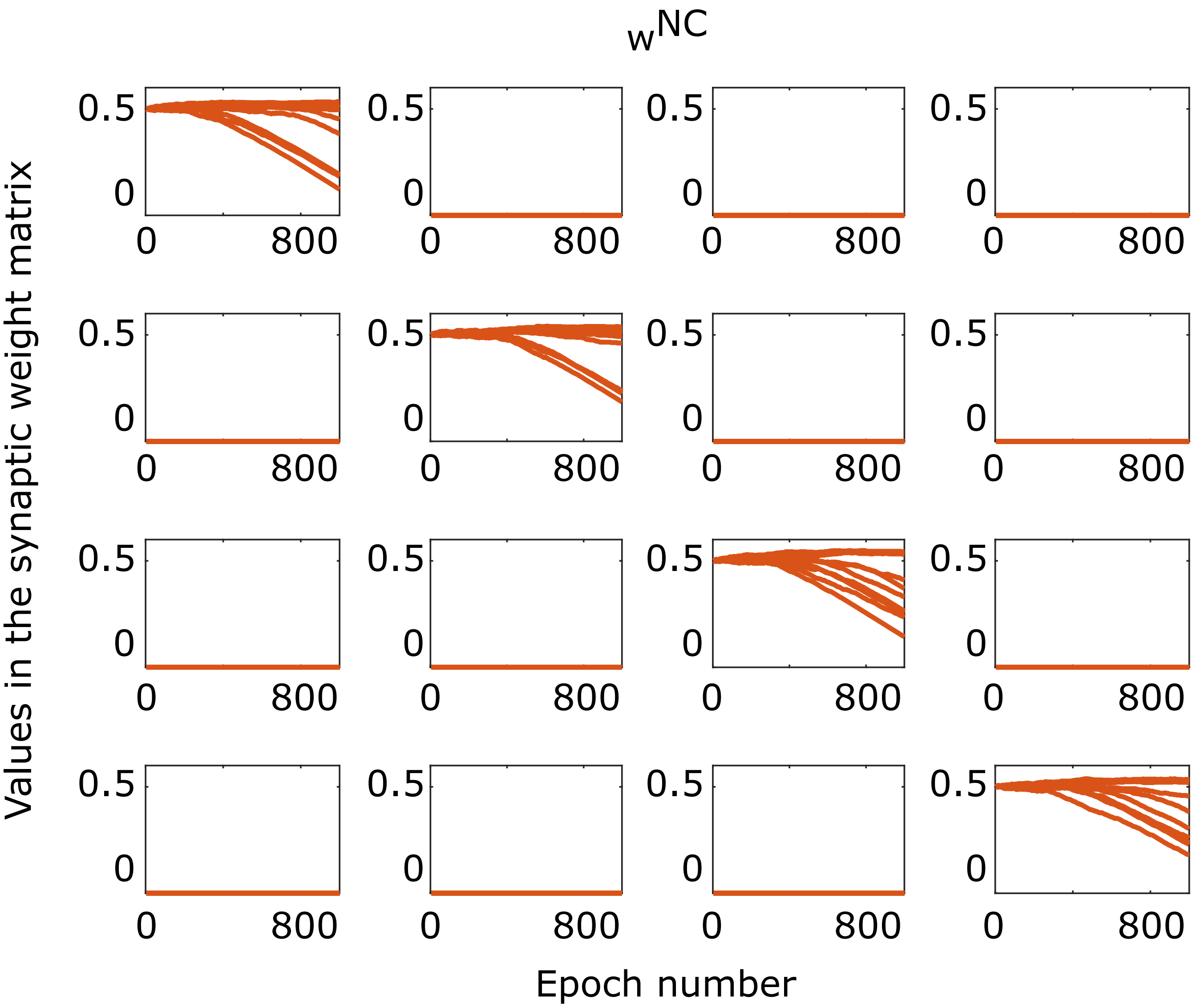

Figure S7. Evolution of the values in the w^{NC} matrix of each individual in the dopamine imbalance group

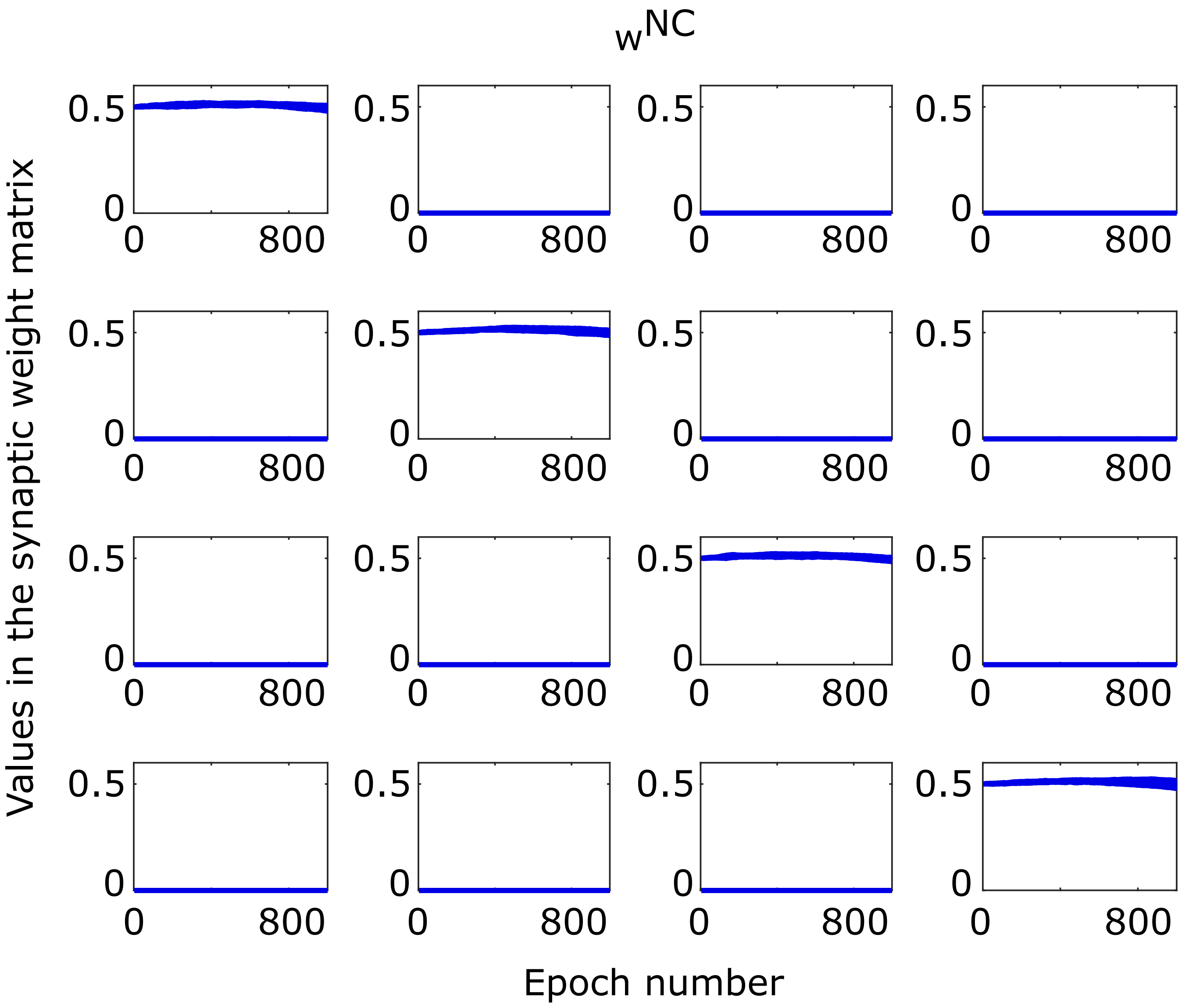

Figure S8. Evolution of the values in the w^{NC} matrix of each individual in the control group
